# Slow viral propagation during initial phase of infection leads to viral persistence in mice

**DOI:** 10.1038/s42003-021-02028-x

**Published:** 2021-04-29

**Authors:** Haifeng C. Xu, Ruifeng Wang, Prashant V. Shinde, Lara Walotka, Anfei Huang, Gereon Poschmann, Jun Huang, Wei Liu, Kai Stühler, Heiner Schaal, Andreas Bergthaler, Aleksandra A. Pandyra, Cornelia Hardt, Karl S. Lang, Philipp A. Lang

**Affiliations:** 1 Department of Molecular Medicine II, Medical Faculty, Heinrich Heine University, Düsseldorf, Germany; 2 Institute of Virology, Medical Faculty, Heinrich Heine University Düsseldorf, Düsseldorf, Germany; 3 Institute of Molecular Medicine I, Proteome research, Medical Faculty, Heinrich-Heine University Düsseldorf, Düsseldorf, Germany; 4 Department of Pediatric Oncology, Hematology, and Clinical Immunology, Medical Faculty, Heinrich Heine University, Düsseldorf, Germany; 5 Molecular Proteomics Laboratory, Biomedical Research Center (BMFZ), Heinrich-Heine-Universität, Düsseldorf, Medical Faculty, Düsseldorf, Germany; 6 CeMM Research Center for Molecular Medicine of the Austrian Academy of Sciences, Vienna, Austria; 7 Institute of Immunology, Medical Faculty, University of Duisburg-Essen, Essen, Germany

**Keywords:** Immune evasion, Infectious diseases

## Abstract

Immune evasion of pathogens can modify the course of infection and impact viral persistence and pathology. Here, using different strains of the lymphocytic choriomeningitis virus (LCMV) model system, we show that slower propagation results in limited type I interferon (IFN-I) production and viral persistence. Specifically, cells infected with LCMV-Docile exhibited reduced viral replication when compared to LCMV-WE and as a consequence, infection with LCMV-Docile resulted in reduced activation of bone marrow derived dendritic cells (BMDCs) and IFN-I production in vitro in comparison with LCMV-WE. In vivo, we observed a reduction of IFN-I, T cell exhaustion and viral persistence following infection of LCMV-Docile but not LCMV-WE. Mechanistically, block of intracellular protein transport uncovered reduced propagation of LCMV-Docile when compared to LCMV-WE. This reduced propagation was critical in blunting the activation of the innate and adaptive immune system. When mice were simultaneously infected with LCMV-Docile and LCMV-WE, immune function was restored and IFN-I production, T cell effector functions as well as viral loads were similar to that of mice infected with LCMV-WE alone. Taken together, this study suggests that reduced viral propagation can result in immune evasion and viral persistence.

## Abstract (web summary)

Using different strains of the lymphocytic choriomeningitis virus (LCMV), Xu, Wang et al. show that a slow viral propagation limits type I interferon (IFN-I) production and viral persistence in mice. This study suggests a reduced viral propagation as a mechanism for immune evasion and viral persistence.

## Introduction

Persistent infection with HIV, HBV, and HCV is a serious global health issue^1,2^. During chronic viral infection, such as with the lymphocytic choriomeningitis virus (LCMV), when the host immune response is compromised and viral antigen is constantly presented, antiviral specific CD8^+^ T cells (CTL) become exhausted. CTL exhaustion can lead to a decrease in cytotoxic function and ability to secrete effector cytokines such as IFN-γ and tumor necrosis factor (TNF)-α^3^. LCMV is a noncytolytic virus, which is widely used as a model system to study chronic infections, virus-induced immunopathology, effector responses, immune tolerance, and T cell exhaustion in mice^4^. LCMV is also used as a tool to uncover factors regulating virus-host interactions^5,6^. LCMV is an ambisense-strand RNA virus composed of two segments. The short segment (S), encodes the preglycoprotein complex (GPC) and the nucleoprotein (NP), whereas the long segment (L) encodes the matrix protein Z and the RNA-dependent RNA polymerase protein L (RdRp)^7^. LCMV enters host cells through binding of the GP to host surface receptors mainly mediated by the surface receptor α-dystroglycan^8^. During LCMV replication, the RdRp binds to NP encapsulated LCMV RNA at the 3’ untranslated region (UTR) and reads towards the 5’UTR. Viral mRNA transcription is terminated by the intergenic hairpin structure formed by the intergenic region (IGR), which is found in both the S- and L-segments^9^. As RdRp continues towards the 5’UTR, a full-length antigenomic S- or L-segment is generated. Accordingly, RdRp uses the antigenome as a template to generate mRNAs for the GPC and Z-protein or full-length S- and L-segments^10^. As indicated by previous studies, viral genomic or antigenomic species can be recognized by the cytosolic RNA sensors RIG-I and/or MAD5, which consequently results in type I interferon (IFN-I) production^11,12^.

Since the initial isolation of LCMV-Armstrong in 1933, many other LCMV strains have been described. LCMV can elicit either acute or chronic infection in immune competent mice in a strain dependent manner. Many key amino acids have been identified to be important for the induction of an LCMV-induced chronic infection. Specifically, GP1 260 L and LP 1079Q in LCMV Clone 13 are thought to be critical for causing viral persistence when compared to LCMV-Armstrong’s GP1 260 F and LP 1079 K^13,14^. Other strains include the LCMV-Docile and LCMV-WE. Mice infected with LCMV Docile exhibit T cell exhaustion and viral persistence, while infection with LCMV-WE results in effective T cell immunity and viral control^4^. However, as demonstrated by sequencing data, both LCMV-WE (acute) and its derivative strain LCMV-Docile (chronic) share the same amino acid at position GP1 260 L and LP 1079 K^15–17^, indicating that additional unknown factors might be present to trigger chronic infection.

IFN-I production following viral infection has an essential antiviral role as mice lacking interferon receptors (IFNAR) are unable to cope with viral infections^18^. Asides from direct antiviral effects through upregulation of interferon stimulated genes (ISG)^19^, IFN-I signalling can inhibit PD-1 expression on CTLs^20^. Furthermore, IFN-I signalling promotes NK inhibitory ligand expression and downregulates NK activating ligand expression on CTLs^20,21^, hence protecting CTLs from NK-cell mediated attack. However, production of IFN-I during chronic LCMV clone 13 infections can trigger the expression of inhibitors of T cell immunity such as PD-L1 and IL-10 ^22,23^. Accordingly, blockade of IFNAR prior to LCMV infection was able to increase T cell immunity and increase LCMV control during chronic viral infection indicating that the timing of IFN-I production is critical for effective antiviral immunity^24,25^. Furthermore, IFN-I production during viral infections can also induce reactive oxygen species (ROS), triggering tissue damage and potentially contributing towards T cell exhaustion^26,27^.

In the present study, the mechanisms underlying chronic infection were investigated using the LCMV-Docile (chronic) and LCMV-WE (acute) strain. Unexpectedly we found that infection with LCMV-WE resulted in increased viral propagation compared to the LCMV Docile during the initial phase of the infection. Higher viral replication caused increased dendritic cell activation and IFN-I production in vitro and in vivo. Mice simultaneously co-infected with both strains exhibited IFN-I production and effective T cell immunity causing reduced viral load compared to the LCMV Docile infected mice.

## Results

### Co-infection with acute and chronic LCMV strains leads to reduced viral load

**Fig. 1:**
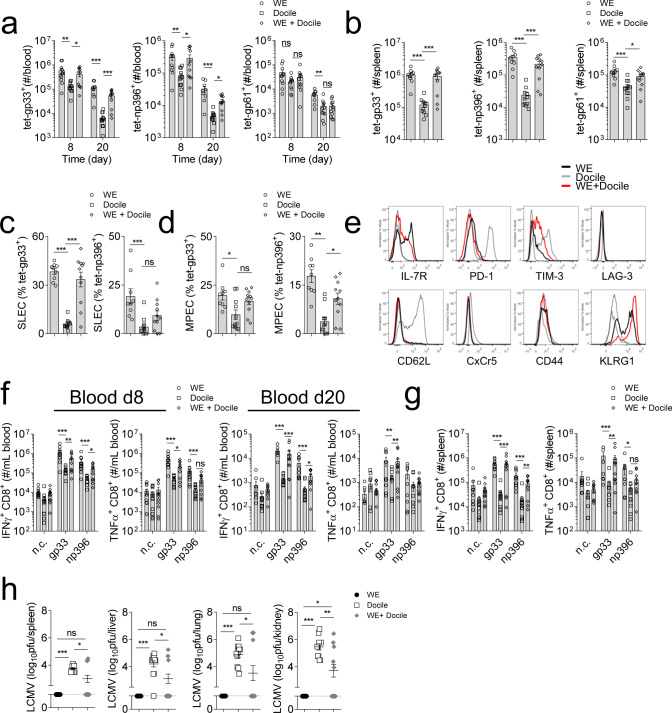
Co-infection with LCMV-WE reverses the immunosuppressive effects of chronic LCMV-Docile viral infection. C57BL/6 mice were infected with LCMV-WE (WE) 2 × 10^4^ pfu, LCMV-Docile (Docile) 2 × 10^4^ pfu, or WE 2 × 10^4^ pfu together with Docile 2 × 10^4^ pfu (WE + Docile) for 20 days. **a**, **b** Mice were sacrificed at the indicated days post-infection and tet-gp33^+^ (CD8^+^), tet-np396^+^ (CD8^+^), and tet-gp61^+^ (CD4^+^) were determined in the **a** blood and **b** spleen tissue (*n* = 9–11). Frequency of **c** short-lived effector cells (SLEC, KLRG1^+^ IL-7R^−^) and **d** memory precursor cells (MEPC, KLRG1^−^, IL-7R^+^) were shown from splenic tet-gp33^+^ and tet-np396^+^ cells (*n* = 9–11). **e** Representative surface molecule FACS blots are shown from splenic tet-gp33^+^ cells (A representative FACS blot of *n* = 9–11 is shown, dotted line represents surface molecule expression of CD19^+^ cells from WE infected hosts). **f**, **g** Mice were sacrificed at the indicated time post-infection and **f** blood cells and **g** single cell suspended splenocytes were re-stimulated with LCMV-specific CD8^+^ T cell epitopes as indicated or left untreated (negative control: n.c.) followed by staining for IFN-γ and TNF-α (*n* = 9–11). **h** at 20 days post-infection, virus titers were determined in the spleen, liver, lung, and kidney tissue (*n* = 9–11). (Error bars show SEM, *p < 0.05, **p < 0.01, ***p < 0.001, and ns indicates statistically not significant between the indicated groups).

Chronic infection results in exhaustion of antigen specific T cells. WT (C57BL/6) animals were challenged intravenously with equal infectious units (2 × 10^4^ pfu) of LCMV-WE (acute) or Docile (chronic). Mice infected with the chronic strain exhibited reduced numbers of LCMV-specific CD8^+^ and CD4^+^ T cells in both blood and spleen tissue when compared to mice infected with the acute strain (Fig. 1a-b). Surprisingly, when the same host was inoculated with both acute (2 × 10^4^ pfu) and chronic (2 × 10^4^ pfu) strains of LCMV, enhanced antigen specific T cell numbers were observed when compared to mice only infected with the chronic strain (Fig. 1a-b). Activated CD8^+^ T cells can be differentiated into short-lived effector T cells (SLEC) or memory precursor cells (MPEC) depending on the surface KLRG1 and IL-7R expression. We observed reduced SLEC (KLRG1^+^ IL-7R^−^) and MPEC (KLRG1^−^ IL-7R^+^) expression in chronically infected hosts when compared to acutely infected counterparts (Fig. 1c-d, Supplementary Fig. 1a). Consistently, co-infection resulted in the rescue of SLEC and MPEC populations when compared to chronically infected animals (Fig. 1c-d). In addition, expression of exhaustion molecules such as PD-1 and TIM-3 was reduced in LCMV-specific CD8^+^ T cells in acute or co-infected hosts when compared to chronically infected hosts (Fig. 1e). The expression of the IL-7R, which maintains T cell survival, was upregulated in acute or co-infected animals compared to mice infected with LCMV Docile alone (Fig. 1e). To evaluate the function of LCMV-specific T cells, blood or splenocytes from infected animals were stimulated with peptides derived from LCMV. We observed a significant increase in IFN-γ and TNF-α producing CD8^+^ T cells in animals with acute- or co- infection, in sharp contrast to their chronically infected counterparts (Fig. 1f-g, Supplementary Fig. 1b-c). We also observed small but significant differences in CD4^+^ T cell responses between the three groups (Supplementary Fig. 1d-e). Neutralizing antibodies were not detectable at day 20 post-infection (Supplementary Fig. 1f). However, while there was no difference in GP binding antibody titers between WE and Docile infected animals 20 days post-infection, co-infected hosts exhibited slightly increased GP binding antibodies (Supplementary Fig. 1g). Consistently, 20 days post-infection, the virus titer was below the detection limit in mice infected with LCMV-WE. However, LCMV was persistent in animals infected with LCMV-Docile (Fig. 1h). LCMV titers in co-infected mice were reduced compared to mice infected with LCMV docile alone (Fig. 1h). Notably, 3 out of 11 co-infected animals were not able to clear LCMV in the spleen. Consistently, T cells from these mice had high expression of exhaustion molecules and low IFN-γ and TNF-α production in response to LCMV epitopes (Supplementary Fig. 2a-b).

### Acute infection results in enhanced DC activation and IFN-I production

**Fig. 2:**
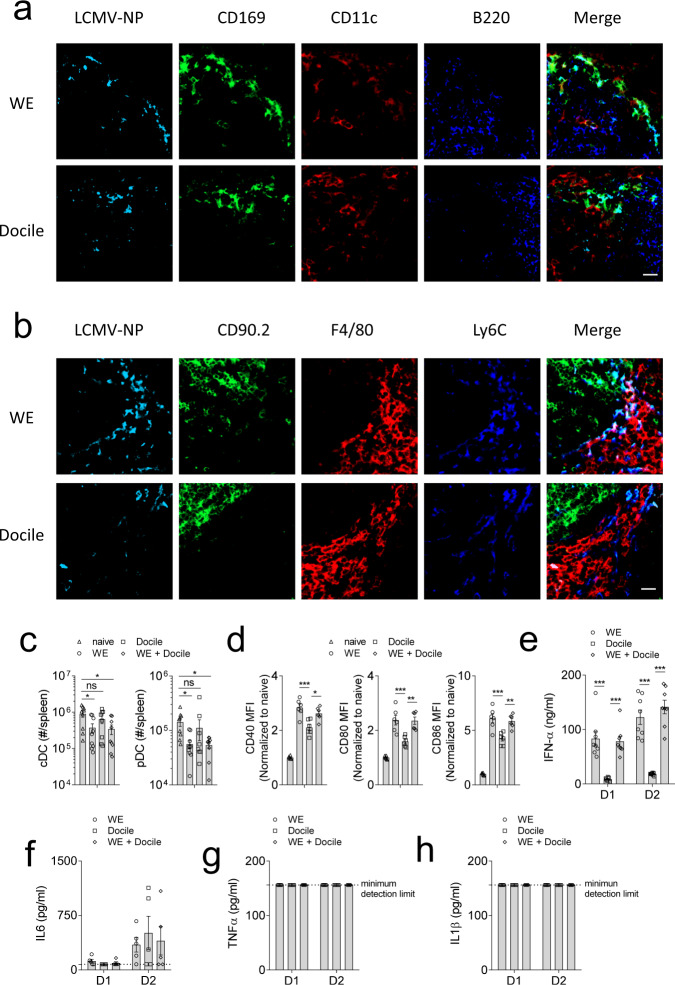
Co-infection with LCMV-WE reverses the immunosuppressive effects of chronic LCMV-Docile viral infection through enhanced DC activation. **a**, **b** C57BL/6 mice were infected with 2 × 10^4^ pfu LCMV-WE (WE), and LCMV-Docile (Docile). At day 2 post-infection, spleen tissues were stained for LCMV antigen NP, CD169, CD11c, and B220 (**a**) or CD90.2, F4/80, and Ly6c (**b**) (representative sections are shown (*n* = 4) scalebar = 20 µm). C57BL/6 mice were infected with LCMV-WE (WE), LCMV-Docile (Docile), or WE together with Docile (WE + Docile). **c**, **d** At day 2 post-infection, numbers of **c** splenic cDC (CD11c^+^ MHC-II^+^) and pDC (B220^+^ Siglec-H^+^) were determined (*n* = 9, gating strategy is shown in Supplementary Fig. 3). **d** Co-stimulatory molecules CD40, CD80, and CD86 were measured on splenic cDC (*n* = 6). **e** Serum IFN-α concentrations were determined at the indicated time post-infection (*n* = 9). **f** Serum IL-6, **g** serum TNF-α, **h** serum IL-1β concentrations were measured at the indicated timepoints (*n* = 5). (Error bars show SEM, *p < 0.05, **p < 0.01, ***p < 0.001, and ns indicates statistically not significant between the indicated groups).

T cell immunity is highly dependent on the ability of antigen presenting cells (APCs). We therefore hypothesized that the differences in T cell activation and viral titres between acute and chronic infection might stem from enhanced activation of APCs. First, we checked the availability of LCMV antigen during the early phase of infection. We observed that LCMV WE and Docile were both distributed in the marginal zone of the spleen (Fig. 2a, b). To investigate dendritic cell (DC) activation, we infected WT animals with the acute or chronic strain of LCMV. Interestingly, LCMV-WE or co-infected animals exhibited reduced numbers of cDCs (CD11c^+^ MHC-II^+^) and pDCs (B220^+^ Siglec-H^+^) (Fig. 2c). Furthermore, we observed that DCs from acute or co-infected mice had increased expression levels of co-stimulatory molecules, such as CD40, CD80, and CD86 when compared to the chronically infected counterparts (Fig. 2d). CD11c expressing cells contribute to IFN-α production following LCMV infection^28^. Consistently, serum from acute and co-infected animals had higher levels of IFN-α post-infection when compared to Docile infected mice (Fig. 2e). However, we did not observe any differences in the proinflammatory cytokines IL-6, TNF-α, and IL-1β (Fig. 2f–h). Next, we infected animals with 2 × 10^4^ or 2 × 10^6^ pfu of WE, Docile, WE together with Docile, and another LCMV strain causing persistent infection: LCMV Clone 13. Docile infected animals showed suppressed IFN-I-mediated and CD8^+^ T cell immunity when compared to other groups (Supplementary Fig. 4a-d). While LCMV-WE and Clone 13 infected mice had effective antiviral T cell immunity and were able to clear LCMV, Docile infected animals could not eliminate the virus and, as a consequence, T cell exhaustion was induced (Supplementary Fig. 4c, d). Consistent with the literature, infection with higher doses of LCMV Clone 13 and Docile, in contrast to LCMV-WE, resulted in T cell exhaustion and virus persistence (Supplementary Fig. 4e, f). Taken together, Docile infected mice demonstrated both impaired IFN-I production and decreased anti-LCMV T cell immunity.

**Fig. 3:**
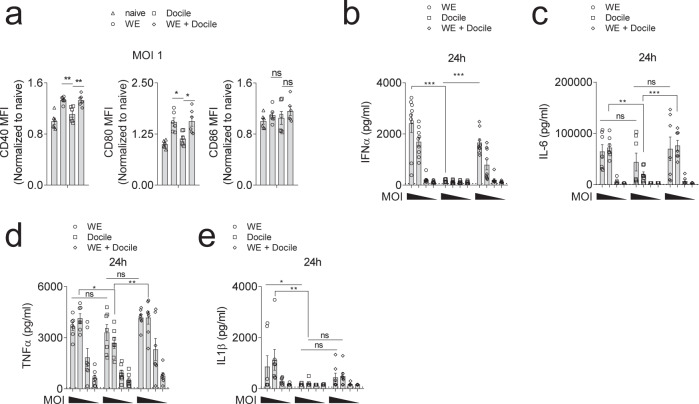
LCMV-Docile failed to activate IFN-I production pathway. GM-CSF induced BMDCs were infected with LCMV-WE, LCMV-Docile, or co-infected at the indicated MOI 10, 1, 0.01 or 0.001. **a** co-stimulatory molecules expression was monitored by flow cytometry 24 h post-infection on CD11c^+^ MHC-II^+^ BMDCs (MOI = 1) (*n* = 6, gating strategy is shown in Supplementary Fig. 5a). **b** IFN-α concentration was determined in the supernatant of LCMV infected BMDCs 24 h post-infection (*n* = 9). **c** IL-6, **d** TNF-α, **e** IL-1β levels were measured in the supernatant of LCMV infected BMDCs 24 h post-infection (*n* = 7, dotted line indicated minimum detection limit). (Error bars show SEM, *p < 0.05, **p < 0.01, ***p < 0.001, and ns indicates statistically not significant between the indicated groups).

To further characterize DC activation during LCMV infection, bone-marrow-derived dendritic cells (BMDCs) were infected with WE or Docile or both strains. Similar to our in vivo findings, we observed increased co-stimulatory molecule expression in WE or co-infected BMDCs when compared to Docile infected BMDCs (Fig. 3a) and reduced IFN-α production (Fig. 3b, Supplementary Fig. 5b). The production of IL-6 and TNF-α was also slightly reduced in Docile infected BMDCs (Fig. 3c, d, Supplementary Fig. 5c, d). Interestingly, the IL-1β production was reduced in LCMV-Docile infected BMDCs when compared to LCMV-WE infected BMDCs (Fig. 3e, Supplementary Fig. 5e–g). Since IFN-I can inhibit mitochondrial respiration^29^, LCMV-WE and co-infected BMDCs had reduced level of oxygen consumption, whereas both uninfected and Docile infected BMDC had high level of oxygen consumption (Supplementary Fig. 5h), indicating a reduced metabolism following IFN-I production in BMDCs. Taken together, these data indicate that LCMV-Docile infection, in sharp contrast to LCMV-WE fails to induce IFN-I production.

### LCMV-WE but not LCMV-Docile infection results in effective PRR activation

**Fig. 4:**
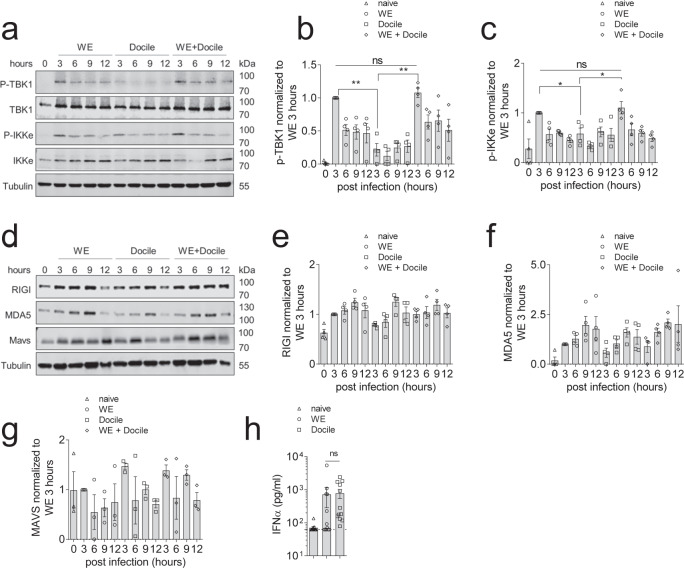
LCMV-WE strain triggers robust pattern recognition receptor (PRR) activation. GM-CSF induced BMDCs were infected with LCMV-WE, LCMV-Docile, or co-infected at a MOI of 1. **a** Whole-cell lysates from LCMV infected BMDCs were blotted for p-TBK1, total TBK1, p-IKKε, total IKKε, and loading control α-Tubulin (one representative blot of *n* = 4 is shown, uncropped blots scans in Supplementary Fig. 6). **b** p-TBK1, **c** p-IKKε quantification was analyzed by first normalizing to the corresponding loading control, followed by standardizing to the 3 h LCMV-WE infected BMDC group (*n* = 4). **d** Whole-cell lysates from infected BMDCs were blotted for RIG-I, MDA5, MAVS, and loading control α-Tubulin at the indicated timepoints post-infection (uncropped blots scans in Supplementary Fig. 8). **e** RIG-I (*n* = 4), **f** MDA5 (*n* = 4), **g** MAVS (*n* = 3) quantification was analyzed by first normalizing to loading control, followed by standardizing to the 3 h LCMV-WE infected BMDC group. **h** JAWSII immature DC cells were transfected with 100 ng of LCMV-WE or LCMV-Docile genomic RNA and 24 h post transfection, IFN-α levels were determined in the supernatant of transfected DC cells (*n* = 12). (Error bars show SEM, *p < 0.05, **p < 0.01, and ns indicates statistically not significant between the indicated groups).

Type I interferon production is mediated by phosphorylated IRF3 and IRF7. IRF3 and IRF7 can be activated by phosphorylated TBK1 and IKKε^30^. When we assessed these signaling pathways using immunoblot analysis, we observed reduced activation of TBK1 and IKKε in Docile infected BMDCs when compared to WE or co-infected BMDCs (Fig. 4a–c). However, NF-κB or MAPK pathway activation was comparable between the different groups (Supplementary Fig. 7). LCMV NP has been reported to interact with RIG-I and MDA5^11^. We hypothesized that Docile-NP affects RIG-I or MAD5 expression and accordingly changes IFN-I production. However, we did not see major differences in RIG-I, MAD5, and MAVS protein expression between acute, chronic or co-infected BMDCs during early course of infection (Fig. 4d–g). Next, we wondered whether the LCMV RNA sequence of WE and Docile are capable of inducing PRR signaling. When BMDCs were transfected with WE or Docile genomic RNA, similar IFN-α production was observed, indicating the failure of IFN-α expression in Docile infected cells was not due to a failure of RIG-I/MDA5 recognition per se (Fig. 4h). Previous studies have suggested that the C-terminal region of the NP protein is important for inhibition of IFN-I production. Specifically, the DIEG motif in the NP C-terminal region was shown to be critical for suppressing the activity of IKKε^31^. However, our sequencing data revealed that both WE and Docile contained this motif (Supplementary Fig. 9). IFN-I production can be amplified through a positive feedback loop via IFNAR signaling^32^. Therefore, we wondered whether LCMV Docile impaired IFNAR signalling. Accordingly, LCMV infected BMDCs were treated with recombinant IFN-α (rIFN-α) and monitored for pSTAT1. As expected, non rIFN-α treated, Docile infected BMDCs showed reduced levels of pSTAT1 when compared to WE or co-infected BMDCs, because of the limited IFN-I production. However, rIFN-α treated Docile infected BMDCs showed increased levels of pSTAT1. The pSTAT1 levels were similar among all rIFN-α treated LCMV infected BMDCs, indicating that Docile did not inhibit the IFNAR positive feedback loop (Supplementary Fig. 10). Taken together, LCMV Docile evaded PRR activation and consequently caused reduced IFN-I production.

### Slow LCMV-Docile propagation leads to impaired innate immune activation

**Fig. 5:**
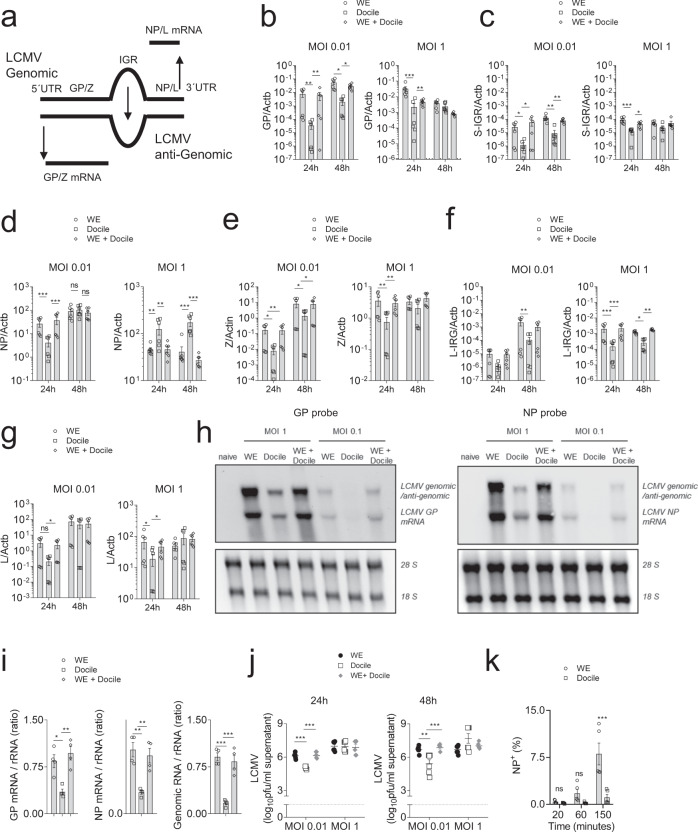
LCMV-Docile exhibited slow propagation compared to the LCMV-WE strain. **a** A schematic of LCMV-S-segment replication is shown. **b**–**g** BHK-21 cells were infected with LCMV-WE, LCMV-Docile, or both at the indicted MOI’s. At 24 h and 48 h post-infection, BHK-21 cellular RNA was isolated and **b** GP RNA, **c** S-IGR RNA, **d** NP RNA, **e** Z RNA, **f** L-IRG RNA, and **g** L RNA were assessed by RT-PCR (*n* = 6). **h** 24 h post-infection, BHK-21 cellular RNA was analysed by northern blot using GP or NP specific probes (one of *n* = 4 representative blot was shown, uncropped scan blots in Supplementary Fig. 11). **i** Ratio of GP mRNA/ribosomal RNA, NP mRNA/ ribosomal RNA and genomic viral RNA/ribosomal RNA were quantified (*n* = 4). **j** Virus titers were determined from LCMV infected BMDC supernatant at 24 h and 48 h post-infection (*n* = 6). **k** BHK-21 cells were infected with LCMV-WE or LCMV-Docile at MOI 0.5, and, at the indicated timepoints post-infection, monensin was added. Sixteen hours later LCMV infected cells were quantified by anti-LCMV-NP staining (*n* = 5, gating strategy is shown in Supplementary Fig. 12). (Error bars show SEM, *p < 0.05, **p < 0.01, ***p < 0.001, and ns indicates statistically not significant between the indicated groups).

IFN-I production through MDA5 and/or RIG-I sensing is important for virus control and CD8^+^ T cell activation during LCMV infection^11,25,33^. While our data suggest that PRR activation by LCMV Docile was reduced, RNA harvested from LCMV Docile was able to induce IFN-I following transfection into DCs. We therefore hypothesized that intracellular RNA levels might be reduced following infection with LCMV Docile. Hence, we designed primers targeting the common region of GPC, IGR, or NP of LCMV-WE and Docile (Fig. 5a). RT-PCR results from LCMV infected BHK-21 cells revealed enhanced GPC and IGR RNA expression in both WE or co-infected groups when compared to Docile infected counterparts (Fig. 5b, c). Notably, small differences in NP RNA expression between WE and Docile infected cells were observed in RT-PCR (Fig. 5d). As NP facilitates binding of the RdRp to viral RNA, these data suggest that a minor difference in NP expression might contribute to the striking difference observed in GP mRNA transcription between the two strains. However, also different RNA expression patterns were observed for the L-segment (Fig. 5e-g). Furthermore, to directly quantify the genomic/antigenomic and mRNA species of LCMV during infection, we harvested cellular RNA from LCMV infected BHK-21 cells and analyzed expression using Northern blotting. Infection with LCMV-WE produced more genomic/antigenomic RNA and mRNA when compared to the Docile strain (Fig. 5h, i). Taken together, these data indicate a reduced replication of LCMV Docile compared to LCMV-WE. Consistently, virus titers in the supernatant of Docile infected cells were reduced compared to LCMV-WE infected cells (Fig. 5j). Next, we wondered whether viral uptake was reduced with the LCMV Docile strain. We infected cells with either LCMV Docile or WE and blocked further viral entry by application of monensin at different timepoints. After 16 h, cell infectivity was determined by assessing NP expression because only small differences in NP mRNA expression were found between both strains. Interestingly, in this assay LCMV-WE infection of BHK-21 cells was highly increased compared to LCMV Docile (Fig. 5k).

**Fig. 6:**
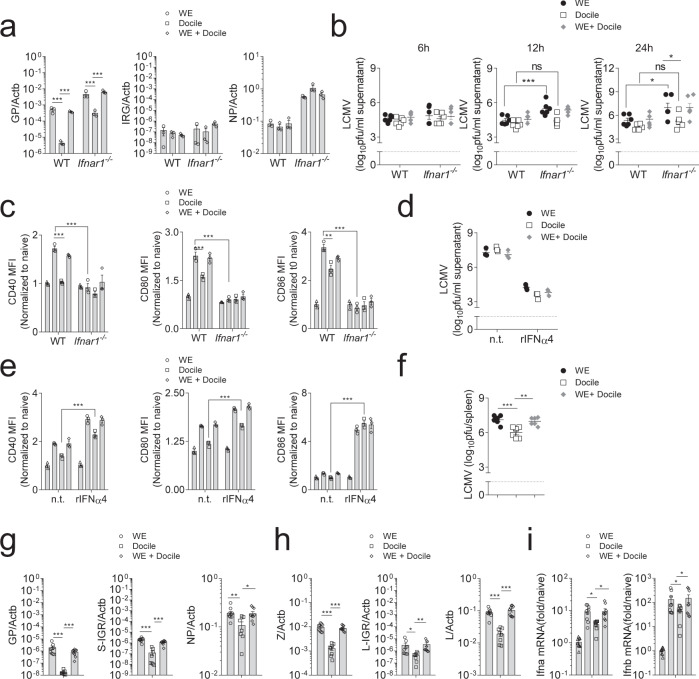
Fast replication of the LCMV-WE strain induced enhanced IFN-I production and promoted DC maturation. **a**–**c** C57BL/6 or IFNAR1 deficient GM-CSF induced BMDCs were infected with LCMV-WE or LCMV-Docile at MOI 1. **a** GP, IGR, and NP region RNA levels were assessed using RT-PCR from infected BMDCs (*n* = 3). **b** Virus titers were determined in the supernatant of LCMV infected BMDC’s at 6, 12, and 24 h post-infection (*n* = 6). **c** Co-stimulatory molecule expression on LCMV infected BMDCs was monitored by flow cytometry 24 h post-infection. **d**, **e** GM-CSF induced BMDCs were infected with LCMV-WE, LCMV-Docile, or co-infected at an MOI 1 in the presence or absence of 100U/ml recombinant IFNα4 (*n* = 3). **d** Virus titers were determined in cell supernatant 24 h post-infection (*n* = 3). **e** Co-stimulatory molecule expression on LCMV infected BMDCs was monitored by flow cytometry 12 h post-infection (*n* = 3). **f**–**i** C57BL/6 mice were infected with LCMV-WE, LCMV-Docile, or both for 2 days. **f** Virus titers were determined in spleen tissues of infected animals (*n* = 6). **g** GP, S-IGR, and NP **h** Z, L-IGR, and L RNA was determined in spleen tissue (*n* = 6). **i** pan-IFN-α and IFN-β mRNA transcripts were measured from spleen tissue (*n* = 6). (Error bars show SEM, *p < 0.05, **p < 0.01, ***p < 0.001, and ns indicates statistically not significant between the indicated groups).

These findings were also observed in BMDCs, where LCMV Docile failed to induce robust IFN-I production. Consistent with our previous results, GPC RNA but not NP RNA expression was significantly increased in WE and co-infected BMDCs when compared to Docile infected BMDCs (Fig. 6a). Interestingly, LCMV titers in the supernatant of BMDCs did not differ despite differences in IFN-I production (Fig. 6b). However, following infection of *Ifnar1*^*−/−*^ BMDCs, increased LCMV titers were detected in the supernatant of LCMV-WE infected cells in comparison to LCMV Docile infected cells (Fig. 6b). Expression of co-stimulatory molecules in BMDCs following LCMV infection was dependent on IFNAR signaling (Fig. 6c). When rIFN-α was added to LCMV infected BMDCs, virus replication was inhibited in all groups (Fig. 6d), and LCMV Docile infected BMDCs upregulated co-stimulatory molecules when compared to the nontreated group (Fig. 6e). Furthermore, in sharp contrast to what we observed 20 days post-infection, during these early timepoints post-infection, reduced LCMV titers were observed in spleen tissues of LCMV-Docile infected animals compared to WE or co-infected mice (Fig. 6f). Moreover, LCMV RNA expression in spleen tissue of LCMV-Docile infected mice was decreased compared to LCMV-WE infected mice (Fig. 6g, h). Consistently, we observed enhanced IFN-I transcripts in spleen tissue of LCMV-WE or co-infected mice when compared to tissues of LCMV-Docile infected mice (Fig. 6i, Supplementary Fig. 13). In order to determine LCMV-WE and Docile RNA expression levels, primers specifically targeting strain specific regions were used. RT-PCR results from LCMV infected BHK-21 cells showed that Docile-GP and NP RNA were reduced in co-infected BHK cells compared to only Docile infected cells (Supplementary Fig. 14a-b), suggesting that LCMV-WE out-competes LCMV Docile in a co-infection setting. Taken together, these data indicate that the LCMV-WE exhibits faster propagation than LCMV-Docile resulting in increased transcription of viral RNA and as a consequence increased PRR activation.

### Slow propagation is mediated by the LCMV-Docile S-Segment

**Fig. 7:**
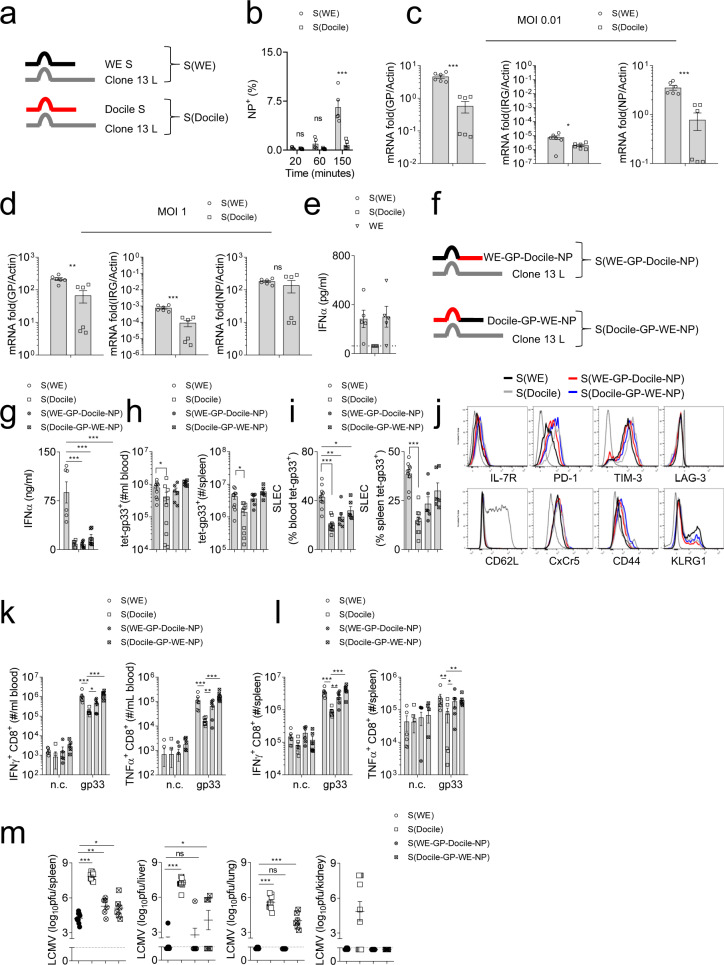
LCMV-Docile S-segment contributes to slow propagation thereby promoting chronic infection. **a** Schematic of chimeric virus S(WE)/L(Clone 13) and S (Docile)/L(Clone 13) is shown. **b** BHK-21 cells were infected with the chimeric virus S(WE)/L(Clone 13) or S (Docile)/L(Clone 13) at MOI 0.5. At the indicated timepoints post-infection, monensin was added. Sixteen hours later, LCMV infected cells were quantified by anti-LCMV-NP staining (*n* = 5). **c**, **d** BHK-21 cells were infected with chimeric virus S(WE)/L(Clone 13) or S (Docile)/L(Clone 13) at MOI 0.01 (**c**), MOI 1 (**d**). At 24 h post-infection, BHK-21 cellular RNA was isolated and GP RNA, S-IGR RNA, and NP RNA were quantified by RT-PCR (*n* = 6). **e** GM-CSF induced BMDCs were infected with chimeric virus S(WE)/L(Clone 13) or S (Docile)/L(Clone 13) or WE WT virus at MOI 1. Twenty four hours post-infection, IFN-α levels were determined in cell supernatants (*n* = 6). **f** A schematic of chimeric virus S(WE-GP-Docile-NP)/L(Clone 13) and S (Docile-GP-WE-NP)/L(Clone 13) is shown. **g**–**m** C57BL/6 mice were infected with 2 × 10^5^ pfu of chimeric virus S(WE)/L(Clone 13), S (Docile)/L(Clone 13), S(WE-GP-Docile-NP)/L(Clone 13), and S (Docile-GP-WE-NP)/L(Clone 13). **g** At day 1 and day 2 post-infection, serum IFN-α levels were determined (*n* = 6). **h** Numbers of tet-gp33^+^ CD8^+^ T cells were determined in blood (left panel), and spleen tissue (right panel) 7 days post-infection (*n* = 6–10). **i** Frequency of short-lived effector cells (SLEC, KLRG1^+^ IL-7R^-^) were quantified in blood (left panel), and spleen (right panel) from LCMV-specific CD8^+^ T cells 7 days post-infection (*n* = 6–10). **j** Expression of T cell surface molecules from spleen tet-gp33^+^ CD8^+^ T cells is shown (representative blots of *n* = 6–10 are shown, dotted line represents surface molecule expression of CD19^+^ cells from S(WE)/L(Clone 13) infected hosts). **k** Blood cells, and **l** single cell suspended splenocytes were re-stimulated with LCMV-specific CD8^+^ T cell epitopes as indicated or left untreated (negative control: n.c.) followed by staining for IFN-γ and TNF-α (*n* = 6). **m** Seven days post-infection, virus titers were determined in the spleen, liver, lung, and kidney tissue (*n* = 6–10). (Error bars show SEM, *p < 0.05, **p < 0.01, ***p < 0.001, and ns indicates statistically not significant between the indicated groups).

We generated chimeric LCMV viruses utilizing the S-segment from LCMV-WE and Docile with the L-segment from Clone 13 (Fig. 7a), here named LCMV-S(WE) and LCMV-S(Docile)^14^. LCMV infection of BHK-21 cells with virus harbouring the S(WE) was faster than infection with LCMV containing the S(Docile) (Fig. 7b). As expected, LCMV-S(WE) showed increased replication in host cells when compared to LCMV-S(Docile) (Fig. 7c, d). Furthermore, LCMV-S(WE) induced IFN-I production in BMDCs in vitro (Fig. 7e). To explore whether Docile-NP plays a role in IFN-I production, we generated mixed chimeric viruses containing WE-GP and Docile-NP or WE-NP and Docile-GP with the L-segment from Clone 13, named LCMV-S(WE-GP-Docile-NP) and LCMV-S(Docile-GP-WE-NP) (Fig. 7f). Infection with LCMV-S(Docile), LCMV-S(WE-GP-Docile-NP), and LCMV-S(Docile-GP-WE-NP) resulted in highly reduced IFN-I production compared with LCMV-S(WE) (Fig. 7g). Only LCMV-S(Docile) infected animals exhibited reduced numbers and function of anti-LCMV CD8^+^ T cells (Fig. 7h–l). However, we did observe a reduction of SLEC in tet-gp33^+^ CD8^+^ T cells from LCMV-S(Docile), LCMV-S(WE-GP-Docile-NP), and LCMV-S(Docile-GP-WE-NP) infected mice when compared to tet-gp33^+^ CD8^+^ T cells from LCMV-S(WE) infected mice (Fig. 7i). Notably, virus strains containing sequences from 2 to 3 different LCMV strains might be attenuated. This is supported by LCMV titers at day 7 post-infection (Fig. 7m). However, LCMV-S(Docile-GP-WE-NP) but not LCMV-S(WE-GP-Docile-NP) infected animals exhibited elevated viral titers when compared to LCMV-S(WE) in liver and lung tissues (Fig. 7m). LCMV-S(Docile) infected animals had elevated viral titers when compared to the other groups (Fig. 7m), but were also able to clear the virus at day 12 post-infection (Supplementary Fig. 15). These data suggest that expression of the Docile S-segment reduced viral propagation, innate and adaptive immune activation, and increased viral load during infection.

## Discussion

In this study we analyzed an immune evasion mechanism during viral infection using the LCMV model system. While the chronic Docile strain failed to induce IFN-I, infection with the acute strain LCMV-WE resulted in IFN-I production. Mechanistically, infection with LCMV- Docile showed reduced PRR activation, which was associated with reduced presence of viral RNA, and reduced viral replication. Co-infection with LCMV strains induced IFN-I production and effective T cell immunity, which resulted in reduced LCMV viral load compared to infection with the chronic LCMV-Docile strain alone.

IFN-I is a critical factor in modulating viral infections. Lack of IFNAR results in excessive viral replication and viral persistence following LCMV infection^18^. Moreover, IFN-I can promote adaptive immunity against viruses. IFN-I is critical for protecting antigen specific T cells from NK-cell-mediated regulation^20,21^. Activated NK cells can target antiviral T cells and contribute to establishment of a chronic viral infection^34–36^. We also observed increased T cell immunity following infection with both LCMV strains. In this setting, IFN-I production post LCMV-WE infection might trigger protection of antiviral T cells, which will recognize both strains. Hence, LCMV load in mice receiving both strains was reduced when compared to animals receiving LCMV Docile alone. However, excess IFN-I can result in expression of ligands triggering T cell exhaustion. PD-1 has been identified as a critical player facilitating loss of T cell function and blockade of PD-1 can restore T cell immunity during chronic viral infection^6^. Interestingly, IFN-I triggers expression of PD-L1 and thus causing persistence of a chronic viral infection. Specifically, it has been shown that the LCMV clone 13 triggered IFN-beta production, which was critical in establishing viral persistence^24^. Notably, NK cell activation during LCMV infection is also triggered by IFN-I, possibly contributing to limited T cell immunity after excess IFN-I production^37^. Viruses can exploit IFN-I to evade adaptive immunity. One method is through excess IFN-I production followed by induction of PD-L1 expression, or by reduced propagation resulting in limited IFN-I production and reduced adaptive immunity. Notably, the kinetics of IFN-I are critical in orchestrating antiviral immunity and minor changes in this might cause profound effects in immunity and viral control^22,23,25^.

Chronic viral infections may avoid immune surveillance during the initial phase of infection through slower viral replication. During Hepatitis C virus infection, plasmacytoid dendritic cells or Kupffer cells can recognize HCV RNA and produce IFN-I^38,39^. Consistently, HCV infected chimpanzees upregulate ISG transcripts. Interestingly, the ISG transcripts peaked around 6-weeks post-infection in chimpanzees who eventually cleared HCV, whereas in chimpanzees who developed a persistent infection, a sustained ISG transcript expression was observed only after 14 weeks^40^. Moreover, during human or chimpanzee HCV infection, the initial HCV titer positively correlated with viral clearance^40,41^. Considering our data, it might be possible that low HCV replication causes reduced APC activation. Accordingly, reduced expression of co-stimulatory molecules might result in reduced or impaired CTL priming, which might contribute to persistence of HCV. Consistently, delayed IFN-I response might promote severe acute respiratory syndrome (SARS)-CoV and SARS-CoV-2 mediated viral pathology^42,43^.

Taken together, our data identify that LCMV Docile exhibits slow viral propagation, reduced innate and adaptive immune activation, and viral persistence, which in part can be reversed by co-infection with an acute strain of LCMV.

## Methods

### Mice

*Ifnar1*^*−/−*^ mice were bred on a C57BL/6 background and maintained under specific pathogen-free conditions. Experiments were performed under the authorization of LANUV in accordance with the German law for animal protection.

### Viruses

LCMV strain WE was originally obtained from F. Lehmann-Grube (Heinrich Pette Institute). LCMV strain Docile was originally obtained from Dr. C.J. Pfau (Troy, NY). Viruses were propagated in L929 cells as previously described^44^. In all experiments, viruses were administered intravenously.

### RNA purification and RT-PCR

Viral RNA obtained from culture supernatant or cells was purified by Trizol according to manufacture’s instructions. GP, IGR, and NP primers were designed to match the WE and Docile S-segment genome. GP, IGR, and NP expression levels were analysed using the iTaq universal SYBR Green 1-Step kit (Bio-Rad). For analysis, the expression levels of all targets were normalized to beta-actin (∆Ct). Common primers for WE and Docile are: GP forward (F): ATCACGAGCATCAAAGCTGTGTA, GP reverse (R): TGAGAGTTGTTGGCTGAGCA, S-IGR-F: TGTAAAAACTATCTGGAAAAGACGC, S-IGR-R: CCCAATGTTGTGACACTCTAAG, NP-F: TCTGATGTCATCAGAACCTTGAC, NP-R: ACCACAAAATGGGCAATTCATAC, Z-F and L-IRG-F: CCAGACACCACCTATCTTGG, Z-R: TCACTCCTCATAGGGAGGTGG, L-IGR-R: AGGTTCAGACTCAAGGGGAA, L-F: ATGCTCACCAACCCAACAAAGAGAA, L-R: TTAGGGTTGACAAAGAAACCAAACT. WE Specific primers are: WE-GP-F: GGACCCACAGAGCGCTATAAGC, WE-GP-R: TGGTACCCCCATTAGATCTCTTAG, WE-NP-F: CCTAGATGAGTTGGCAACAA, WE-NP-R: TCTGGGTGAGTTAGCTACAG, Docile specific primers are: Docile-GP-F: AGATGCACAGAGTGCTCTGAGT, Docile-GP-R: CGGCACTCCCATCAAGTCTCTCAA, Docile-NP-F: TCTGGGTGAGTTAGCTACAG, Docile-NP-R: GCTAAAGGATAAACACCCAGTTCTG. Actb-F: GGCTGTATTCCCCTCCATCG, Actb-R: CCAGTTGGTAACAATGCCATGT, panIFNa-F: TCTGATGCAGCAGGTGGG, panIFNa-R: AGGGCTCTCCAGACTTCTGCTCTG, IFNb1-F: CAGCTCCAAGAAAGGACGAAC, IFNb1-R: GGCAGTGTAACTCTTCTGCAT.

### Viral sequence analysis

Viral RNA was reverse transcribed utilizing the ProtoScript^®^II First Strand cDNA Synthesis Kit (New England Biolabs GmbH, Frankfurt, Germany) and random primers according to the manufacturer’s protocol. LCMV-WE and LCMV-Docile cDNA was amplified with Taq DNA Polymerase (Qiagen, Hilden, Germany) and overlapping LCMV-specific primer pairs to cover the S-segment and L-segment of both LCMV strains. Purified PCR products (Qia Quick PCR purification Kit) were sequenced utilizing the Big Dye^TM^ Terminator v1.1 Cycle Sequencing Kit (Applied Biosystems^TM^) and analyzed on a Prism Genetic Analyzer 3130-16 (Applied Biosystems^TM^). Terminal Sequences (5’ and 3’ ends) of S- and L-segments were obtained from viral RNA utilizing the Superscript^TM^ IV First-Strand Synthesis System (Thermo Fisher Scientific) according to the manufacturer’s protocol and gene specific primer for first-strand synthesis, followed by dA tailing and second strand synthesis with oligo-T-tailed primers. The second strand synthesis product was PCR amplified utilizing Q5^®^ Hot Start High-Fidelity DNA polymerase (New England Biolabs GmbH, Frankfurt, Germany) oligo -T-tailed primers and nested gene specific primers. Purified PCR products were sequenced with nested gene specific primers as described above.

### Bone-marrow-derived dendritic cell (BMDC) generation

BMDCs were generated as previously described^45^. Briefly, 2 million bone marrow cells were cultured in nontissue culture treated dishes in the presence of GM-CSF (40 ng/ml). On day 3, day 6, and day 8, fresh medium containing GM-CSF was added into the BMDC culture. Experiments were preformed at day 10 post GM-CSF culture.

### Flow cytometric analysis

Experiments were performed using a FACS Fortessa and analyzed using FlowJo software. For dendritic cell staining, singly suspended cells were incubated with antibodies (anti-CD19, CD3, CD8, CD11c, MHC-II, B220, Siglec-H, CD40, CD80, and CD86) for 30 min at 4 °C. Tetramer and intracellular cytokine staining were performed as described previously^20^. For tetramer staining, singly suspended cells were incubated with tetramer-gp33 or tetramer-gp34 (CD8) for 15 min at 37 °C. After incubation, surface antibodies (anti-CD8, IL-7R, KLRG1, CD44, CD62L, PD-1, TIM-3, LAG-3, CXCR5) were added for 30 min at 4 °C. For intracellular cytokine re-stimulation, singly suspended cells were stimulated with LCMV-specific peptides gp33, np396, and gp61 for 1 h. Brefeldin A (eBiocience) was added for another 5 h’ incubation at 37 °C followed by staining with anti-CD8/anti-CD4, anti-IFN-γ, and anti-TNF-α.

### Northern blot analysis

Five micrograms of RNA were separated on a denaturing 1% agarose gel and capillary blotted overnight onto a positively charged nylon membrane by using 20× SSC (3 M NaCl, 300 mM tri-sodium-citrate). The RNA was UV crosslinked to the membrane, the large and small rRNAs were marked, and the membrane was washed twice with dH_2_O. After 2 h of prehybridization with 10 ml 1× DIG Easy Hyb hybridization solution (Roche) at 55 °C, the membrane was hybridized with specific digoxigenin (DIG)-labeled GP and NP-PCR probes (DIG-11-dUTP alkali-labile; Roche). Following the overnight hybridization at 55 °C, the membrane was washed twice with dH_2_O and twice with stringent wash buffer I (2× SSC, 0.1% SDS) at room temperature, followed by two 20 min washing steps in stringent wash buffer II (0.2× SSC, 0.1% SDS) at 68 °C. After two additional washing steps with dH_2_O, the membrane was incubated in maleic acid buffer (0.1 M maleic acid, 150 mM NaCl, pH 7.5) and blocked with 1× blocking solution (Roche) dissolved in maleic acid buffer for 45 min. Anti-Digoxigenin-AP, Fab fragments (Roche) were diluted 1:20,000 in 1× blocking solution and incubated for 1 h at room temperature (RT). The membrane was washed three times with maleic acid buffer (10 min each), and the RNA bands were visualized by using CDP-Star for chemiluminescent reactions (1:100 in AP buffer [0.1 M Tris HCl, 0.1 M NaCl, pH 9.5]; Roche). The blots were visualized using the Lumi-Imager™ F1 (INTAS).

### Viral uptake assay

BHK-21 cells were kept on ice together with LCMV virus at MOI 0.5 for 1 h. Then BHK-21 cell and LCMV virus mixture were incubated at 37 °C. At 20, 60, and 150 min post incubation, monensin was added. After overnight incubation, LCMV infected cells were quantified by flow cytometry using anti-LCMV NP antibodies (clone VL-4, generated in-house).

### LCMV antibody assays

Analysis of LCMV glycoprotein specific antibodies and neutralizing LCMV antibodies were performed as previously described^46^.

### Histology

Histological analysis of snap frozen tissue was performed as previously described^47^. Antibodies against CD169 (Bio-Rad), CD11c, B220, CD90.2, F4/80, Ly6C (ebioscience), donkey-anti-rat secondary antibody (jackson immunoresearch), and self-made anti-LCMV monoclonal antibody (Clone: VL-4), were used. Images were acquired by ZEISS Axio Observer Z1.

### Immunoblotting

Cells were lysed with zjr RIPA lysis buffer supplemented with a protease inhibitor cocktail (Sigma, P8340). Protein concentration in cell lysates was measured using the Advanced Protein assay Reagent: 5 × Concentrate (Advanced, ADV01). Equal amounts of cell lysate were subjected to separation by SDS–PAGE, then transferred to Nitrocellulose Blotting Membrane (GE Healthcare Life Sciences, 10600002). After transfer, blotted membrane was blocked with freshly prepared Odyssey^®^ Blocking Buffer (PBS), diluted in PBS (1:1) for 30–60 min. Primary antibody was diluted in blocking buffer and the membranes were incubated overnight at 4 degrees. IRDye 680RD (LI-COR, 925-68070) or IRDye^®^ 800CW (LI-COR, 925-32211) secondary antibodies were used for visualization using the LI-COR Odyssey imager FC 2800.

### Statistics and reproducibility

Data are expressed as mean ± S.E.M. D’Agostino & Pearson or Shapiro-Wilk test was used for a normality test. For analysis of statistical significance between two groups, a students *t*-test was used. For analysis of statistical significance between multiple groups, a one-way ANOVA was used. For analysis of multiple timepoint experiments, two-way ANOVA with an additional Bonferroni post-test or Fisher’s LSD test was used. Mann-Whitney or Kruskal-Wallis nonparametric tests were applied when samples were not normally distributed. Linear regression was used to model the relationship between T cell exhaustion and viral persistence. *P* < 0.05 was considered to be statistically significant.

### Reporting summary

Further information on research design is available in the Nature Research Reporting Summary linked to this article.

## Supplementary information

Supplementary Information

Description of Additional Supplementary Files

Supplementary Data 1

Reporting Summary

## Data Availability

All relevant data are available from the authors upon request from P.Lang (langp@uni-duesseldorf.de). Source data for the main figures are presented in Supplementary Data 1.
