# Supplementary material for: Slow viral propagation during initial phase of infection leads to viral persistence in mice
Source: Commun Biol. 2021 Apr 29;4:508. doi: 10.1038/s42003-021-02028-x (PMC8084999; doi:10.1038/s42003-021-02028-x)
Supplement: Supplementary file 1 — Supplementary Information [file 42003_2021_2028_MOESM1_ESM.pdf]

**Title: Slow viral propagation during initial phase of infection leads to viral persistence in mice**

Haifeng C. Xu1*, Ruifeng Wang1*, Prashant V Shinde1, Lara Walotka2, Anfei Huang1, Gereon Poschmann3, Jun Huang1, Wei Liu1,4, Kai Stühler3,5, Heiner Schaal2, Andreas Bergthaler6, Aleksandra A. Pandyra1,4, Cornelia Hardt7, Karl S. Lang7, Philipp A. Lang1#

1Department of Molecular Medicine II, Medical Faculty, Heinrich Heine University, Universitätsstr. 1, 40225 Düsseldorf, Germany, 2Institute of Virology, Medical Faculty, Heinrich Heine University Düsseldorf, D-40225 Düsseldorf, Germany. 3Institute of Molecular Medicine I, Proteome research, Medical Faculty, Heinrich-Heine University Düsseldorf, Universitätsstraße 1, 40225 Düsseldorf, Germany, 4Department of Pediatric Oncology, Hematology, and Clinical Immunology, Medical Faculty, Heinrich Heine University, Universitätsstr. 1, 40225 Düsseldorf, Germany, 5Molecular Proteomics Laboratory, Biomedical Research Center (BMFZ), Heinrich-Heine-Universität, Düsseldorf, Medical Faculty, Duesseldorf, Germany, 6CeMM Research Center for Molecular Medicine of the Austrian Academy of Sciences, Lazarettgasse 14 AKH BT25.3, 1090 Vienna, Austria, 7Institute of Immunology, Medical Faculty, University of Duisburg-Essen, Essen, Germany.

*These authors contributed equally to this work.
#Address correspondence to PAL: langp@uni-duesseldorf.de

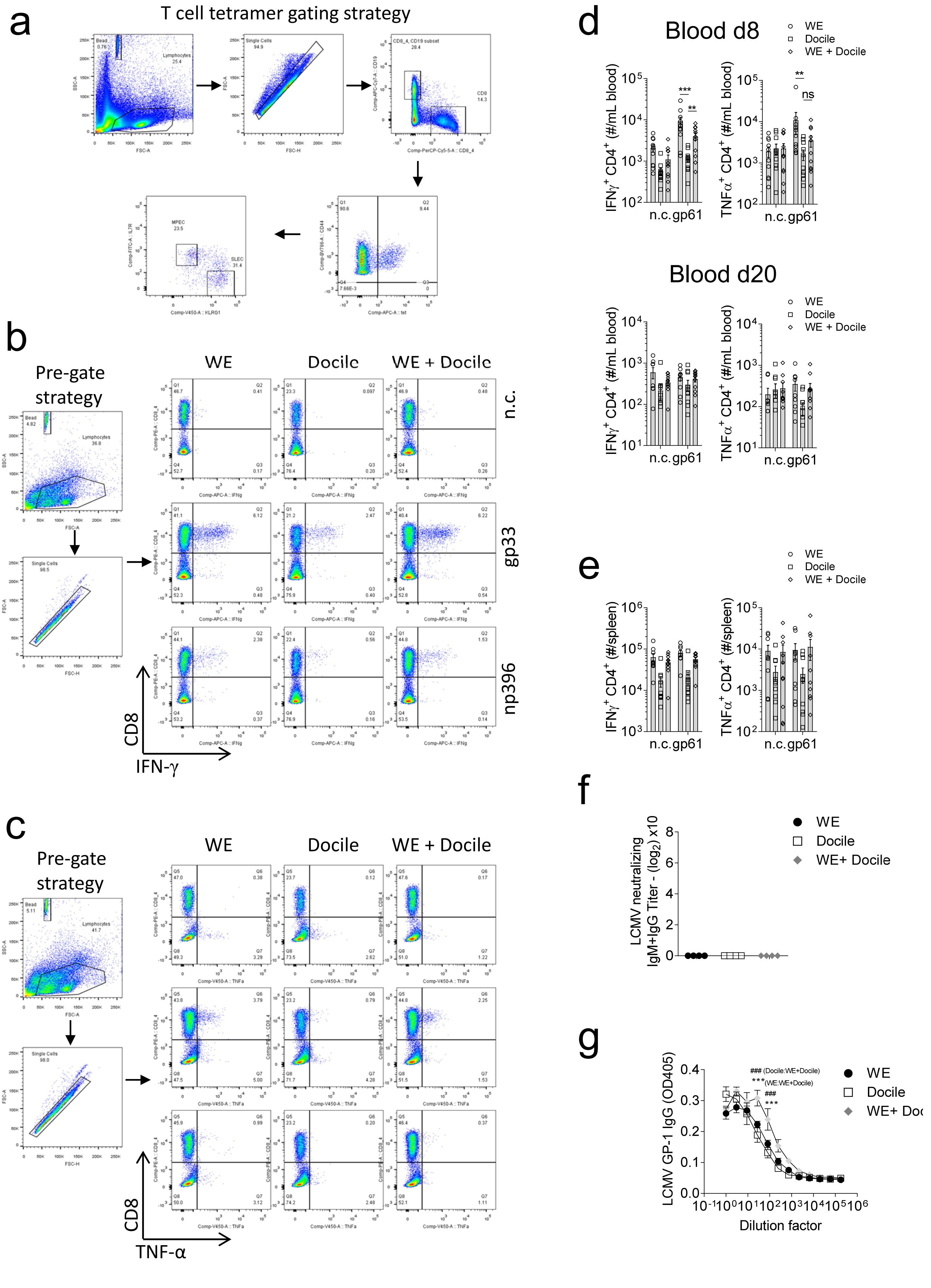
a
T cell tetramer gating strategy
Lymphocytes
Single Cells
CD8
MPEC
SLEC
b
Pre-gate strategy
WE
Docile
WE + Docile
n.c.
gp33
np396
CD8
IFN-γ
c
Pre-gate strategy
WE
Docile
WE + Docile
CD8
TNF-α
d
Blood d8
WE
Docile
WE + Docile
IFNγ+ CD4+ (#/mL blood)
TNFα+ CD4+ (#/mL blood)
n.c. gp61
***
**
ns
Blood d20
e
IFNγ+ CD4+ (#/spleen)
TNFα+ CD4+ (#/spleen)
f
LCMV neutralizing IgM+IgG Titer - (log2) x10
WE+ Docile
g
LCMV GP-1 IgG (OD405)
Dilution factor
### (Docile:WE+Docile)
***(WE:WE+Docile)

**Supplementary Figure 1: TNF-α and IL-β production *in vivo* is limited during LCMV infection.** C57BL/6 mice were infected with LCMV-WE, LCMV-Docile, or co-infected. **(a)** T cell tetramer gating strategy of Figure 1a-d. Representative FACS blot of Figure 1F are shown for IFN-γ **(b)** and **(c)**TNF-α staining. At the indicated time points post-infection, **(d)** blood cells, **(e)** singly cell suspended splenocytes were re-stimulated with LCMV-specific $CD4^+$ T cell epitopes as indicated followed by staining for IFN-γ and TNF-α (n=9-11). **(f)** Serum neutralizing antibodies against LCMV were determined at day 20 post-infection (n=4). **(g)** Serum LCMV GP specific binding antibodies were quantified at day 20 post-infection (n=4). (Error bars show SEM, **p <0.01, ***p < 0.001, ###p<0.001, and ns indicates statistically not significant between the indicated groups).

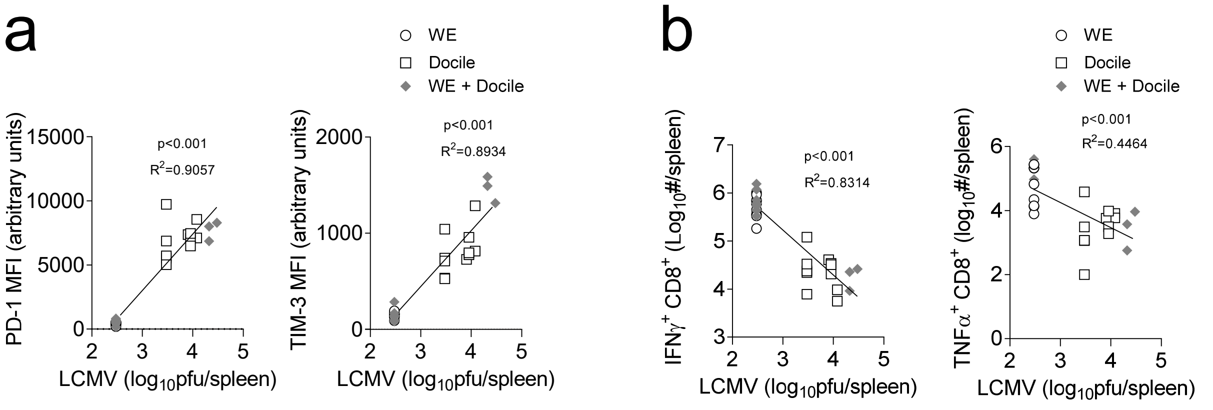


**Supplementary Figure 2: Viral presence positively correlates with T cell exhaustion.**
**(a)** PD-1 or TIM3 expression of splenic tet-gp33 from Figure 1E were plotted against spleen LCMV titers from Figure 1H. **(b)** IFN-γ or TNF-α producing $CD8^+$ T cells in response to LCMV gp33 peptides (Figure 1g) were plotted against spleen LCMV titers (Figure 1h). ($p<0.001$ indicates slope significantly non-zero).

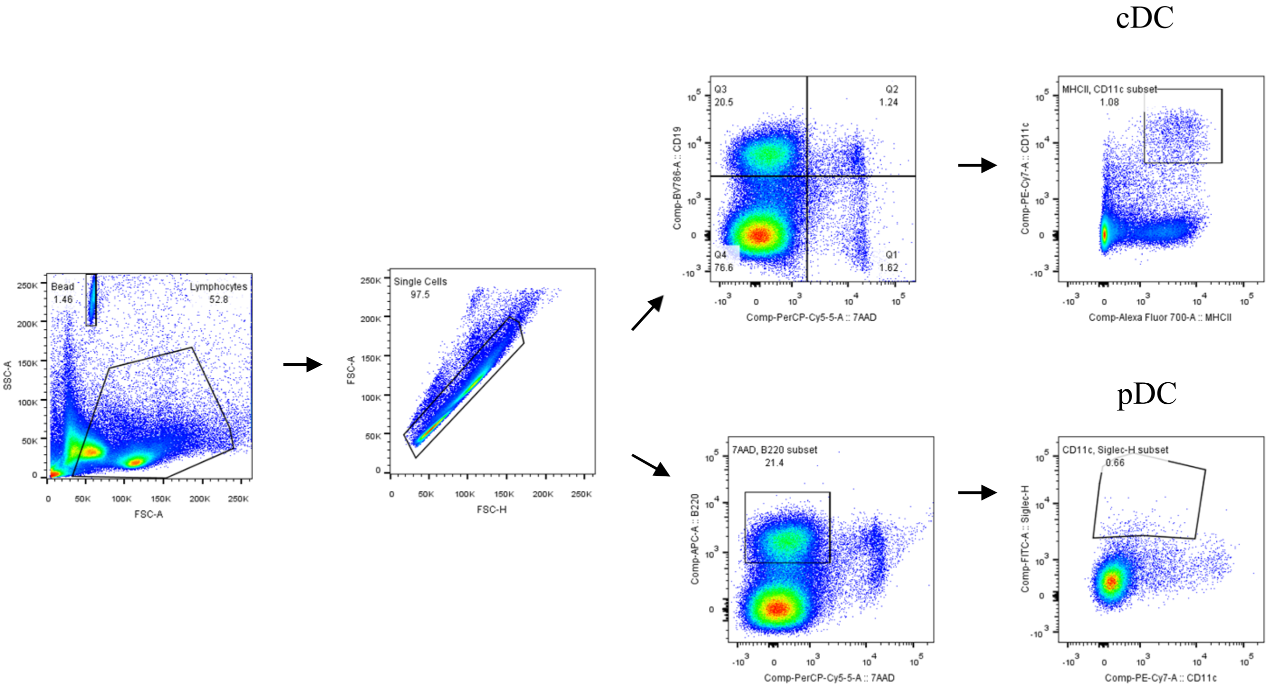


**Supplementary Figure 3: cDC and pDC gating strategy of Figure 2c**

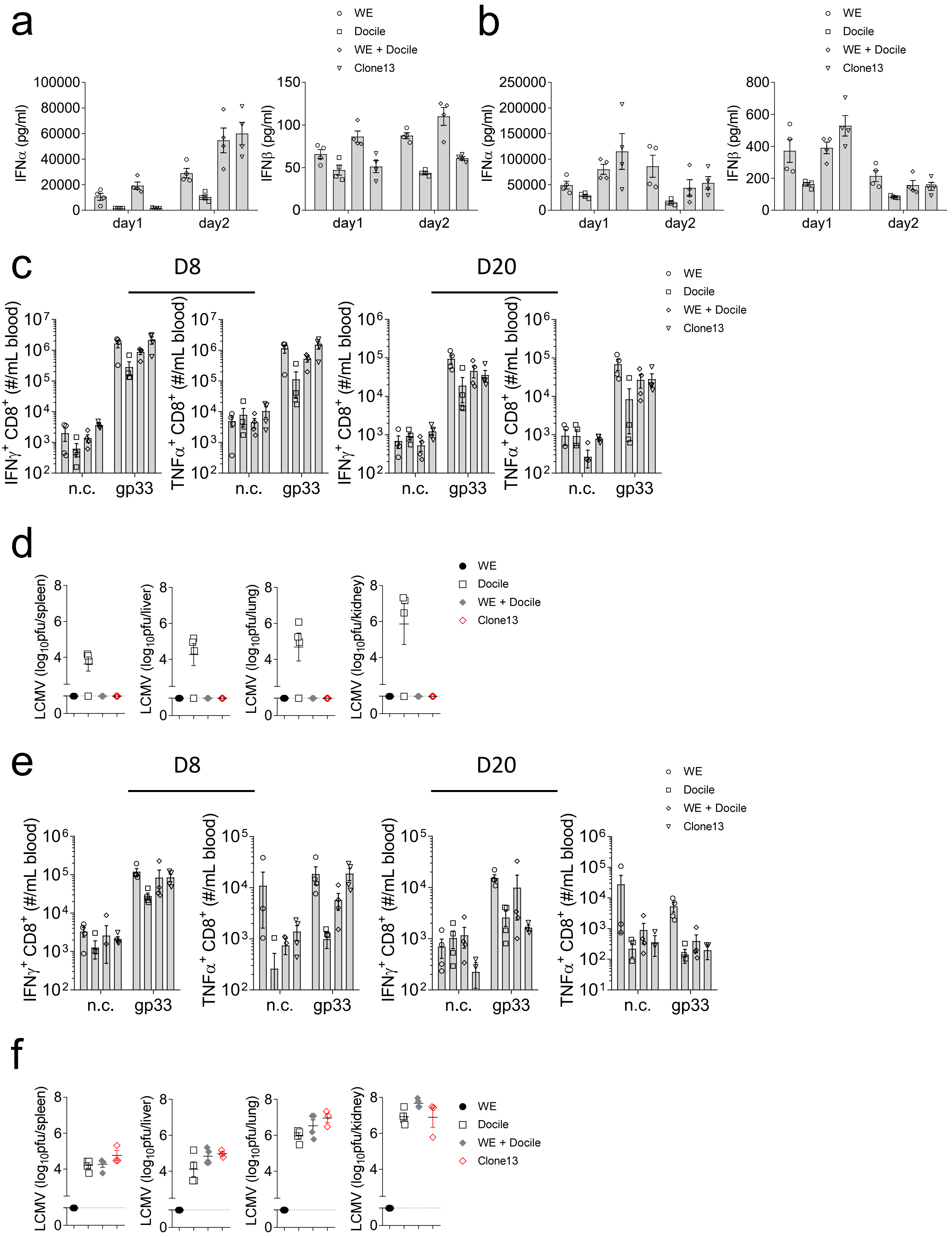
a
b
c
d
e
f
WE
Docile
WE + Docile
Clone13
IFNα (pg/ml)
IFNβ (pg/ml)
day1
day2
D8
D20
IFNγ+ CD8+ (#/mL blood)
TNFα+ CD8+ (#/mL blood)
n.c.
gp33
LCMV (log10pfu/spleen)
LCMV (log10pfu/liver)
LCMV (log10pfu/lung)
LCMV (log10pfu/kidney)

**Supplementary Figure 4: LCMV-Docile infection led to defective IFN-I production.**

C57BL/6 mice were infected with $2x10^4$ pfu **(a+c+d)** or $2x10^6$ pfu **(b+e+f)** LCMV-WE, LCMV-Docile, co-infected WE and Docile or Clone 13. **(a-b)** At indicated days post-infection, serum IFN-α (left panel) or IFN-β (right panel) were quantified (n=4). **(c+e)** At day 8 or day 20 post-infection, blood cells were re-stimulated with LCMV-specific $CD8^+$ T cell epitopes as indicated or left untreated (negative control: n.c.) followed by staining for IFN-γ and TNF-α (n=3-4). **(d+f)** At day 20 post-infection, virus titers were determined in the spleen, liver, lung, and kidney tissue (n=3-4).

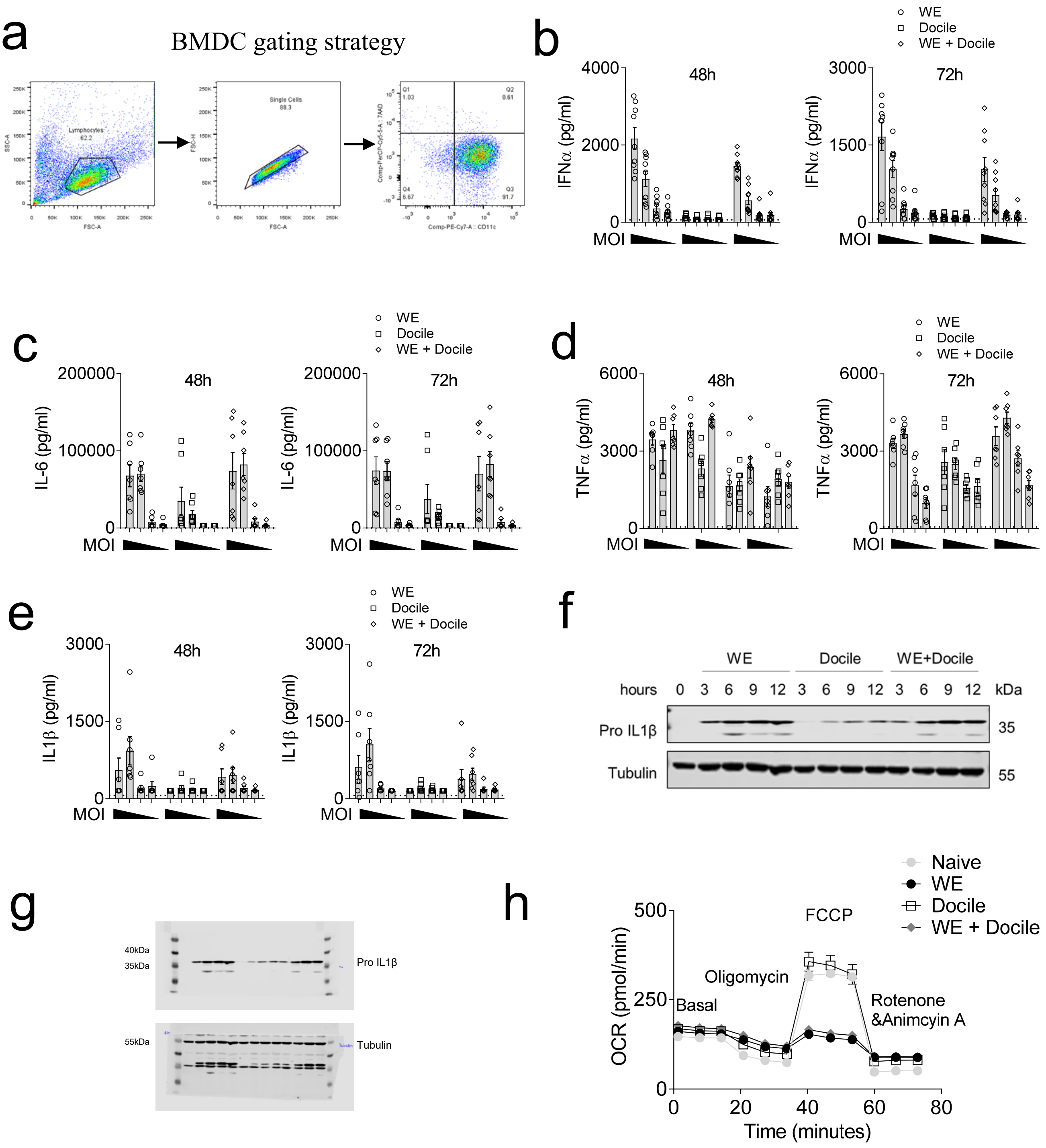


**Supplementary Figure 5: LCMV-WE infection promotes enhanced dendritic cell activation.**
**(a)** BMDC gating strategy of Figure 3a. **(b)** GM-CSF induced BMDCs were infected with LCMV-WE or LCMV-Docile, or co-infected at the indicated MOI's. IFN-α concentration was determined in the supernatant of infected BMDCs 48h and 72h post-infection (n=9). **(c-e)** GM-CSF induced BMDCs were infected with LCMV WE or Docile, or co-infected at the indicated MOI's, 48h, and 72h post-infection and **(c)** IL-6, **(d)** TNF-α, **(e)** IL-1β levels were determined in the supernatant of infected BMDCs (n=7). **(f)** GM-CSF induced BMDCs were infected with LCMV-WE, LCMV-Docile, or co-infected at MOI 1. At the indicated time points, IL-1β was determined by immunoblot analysis (A representative of n=4 immunoblots is shown). **(g)** uncrop scan of Western Blots in **(f)**, **(h)** GM-CSF induced BMDCs were infected with LCMV-WE or LCMV-Docile, or co-infected at a MOI =10. 24 h post-infection, the oxygen consumption rate (OCR) was determined in real time after the addition oligomycin, FCCP, and antimycin-A/rotenone (n=9-11).

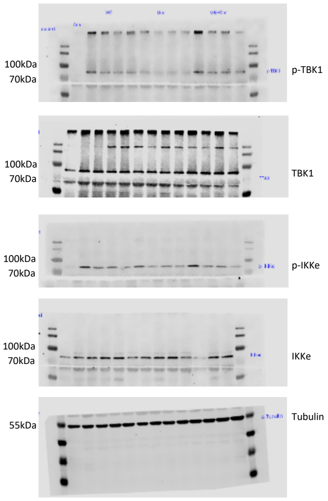


**Supplementary Figure 6: Uncropped scans of Western blots in Fig. 4a**

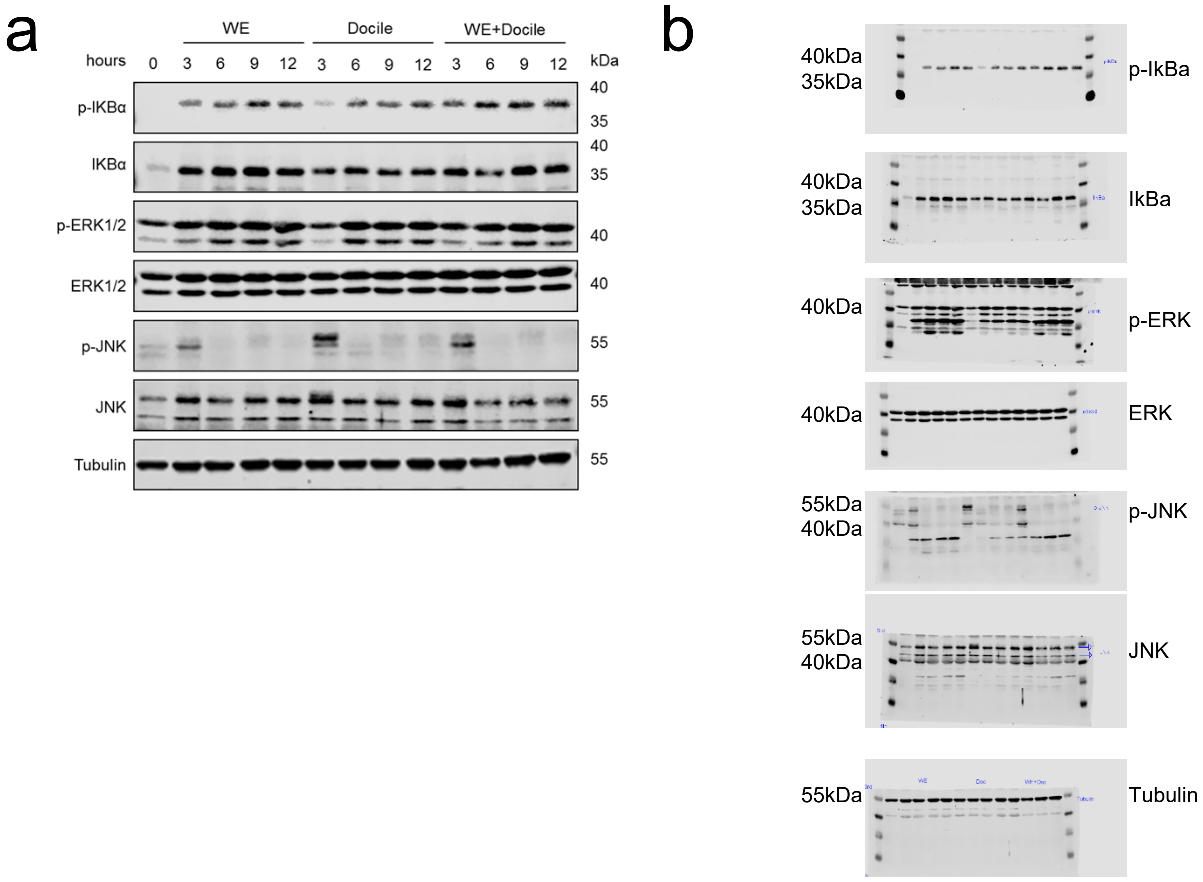


**Supplementary Figure 7: Both LCMV-WE and LCMV-Docile infections induced NF-κB and MAP kinase activation.**

**(a)** GM-CSF induced BMDCs were infected with LCMV-WE, LCMV-Docile, or co-infected at a MOI=1. p-IKBα, total IKBα, pERK1/2, ERK1/2, p-JNK, total JNK, and their loading control Tubulin were assessed by immunoblot analysis at the indicated time points (one of n=4 representative blot was shown). **(b)** Uncropped scans of Western blots in **(a).**

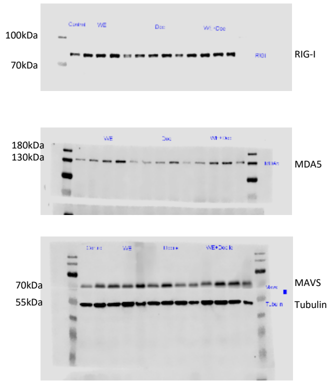


**Supplementary Figure 8: Uncropped scans of Western blots in Fig. 4d**

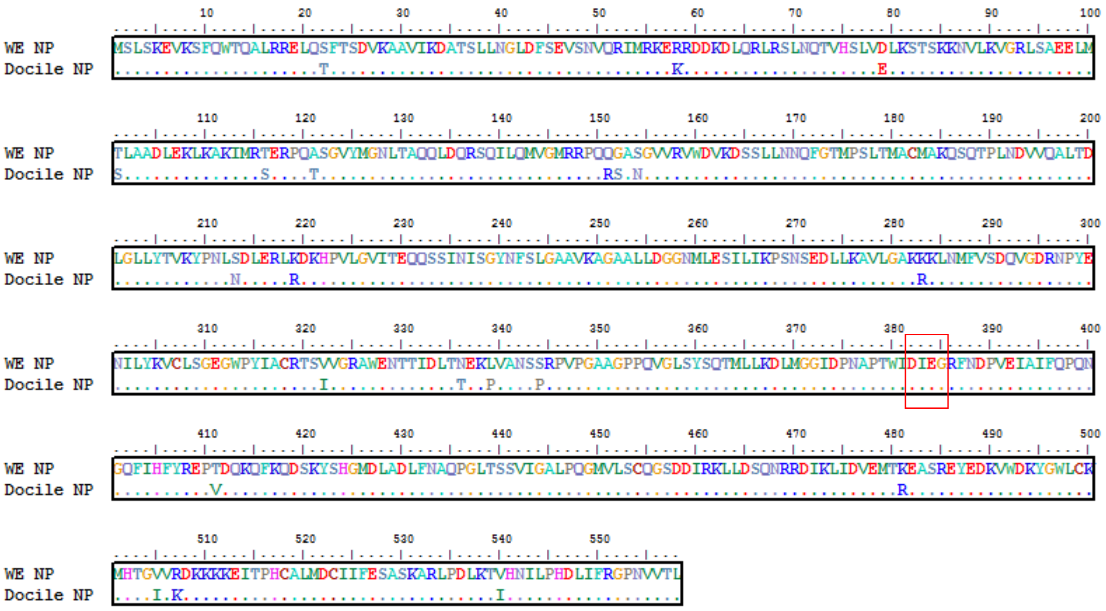

**Supplementary Figure 9: LCMV-WE and LCMV-Docile NP share the same DIEG motif**
NP Amino acids sequence were between WE and Docile Strain. The DIEG motif is highlighted in red box.

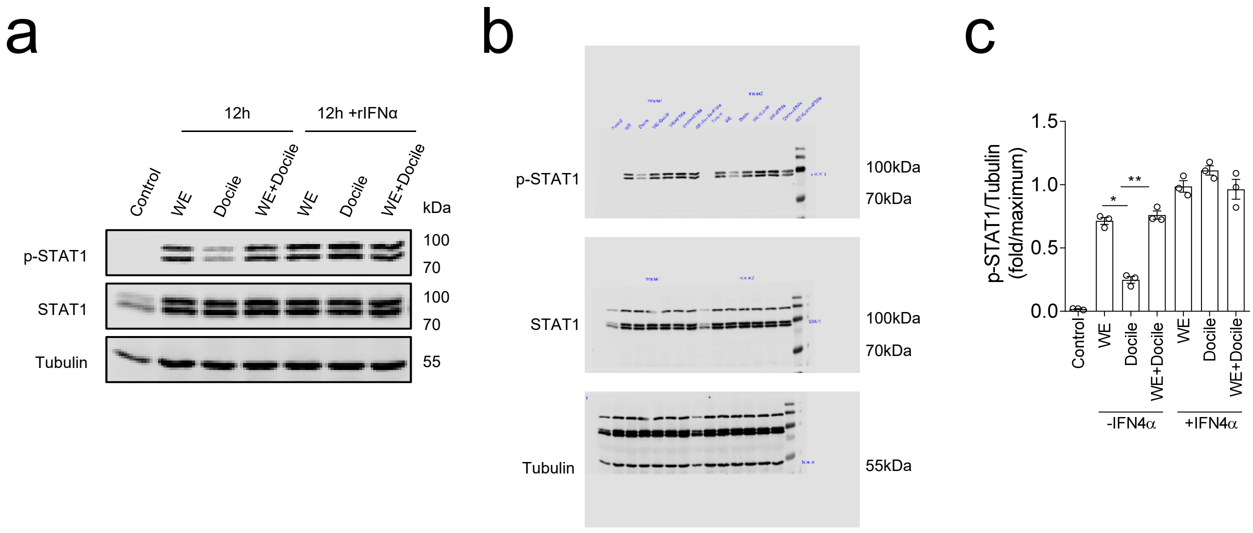


**Supplementary Figure 10: LCMV-WE and LCMV-Docile did not block IFNAR signalling.**
GM-CSF induced BMDCs were infected with LCMV-WE, LCMV-Docile, or co-infected at MOI 1. 12 h post-infection, BMDCs were treated with recombinant IFNα4 at 100U/ml and 30 minutes later, (a) p-STAT1, total STAT1 were measured by immunoblotting (A representative blot of n=3 is shown). (b) Uncropped scans of Western blots in **(a)**. **(c)** Quantification of (a) is shown (n=3). (Error bars show SEM, *p<0.05, **p <0.01 between the indicated groups).

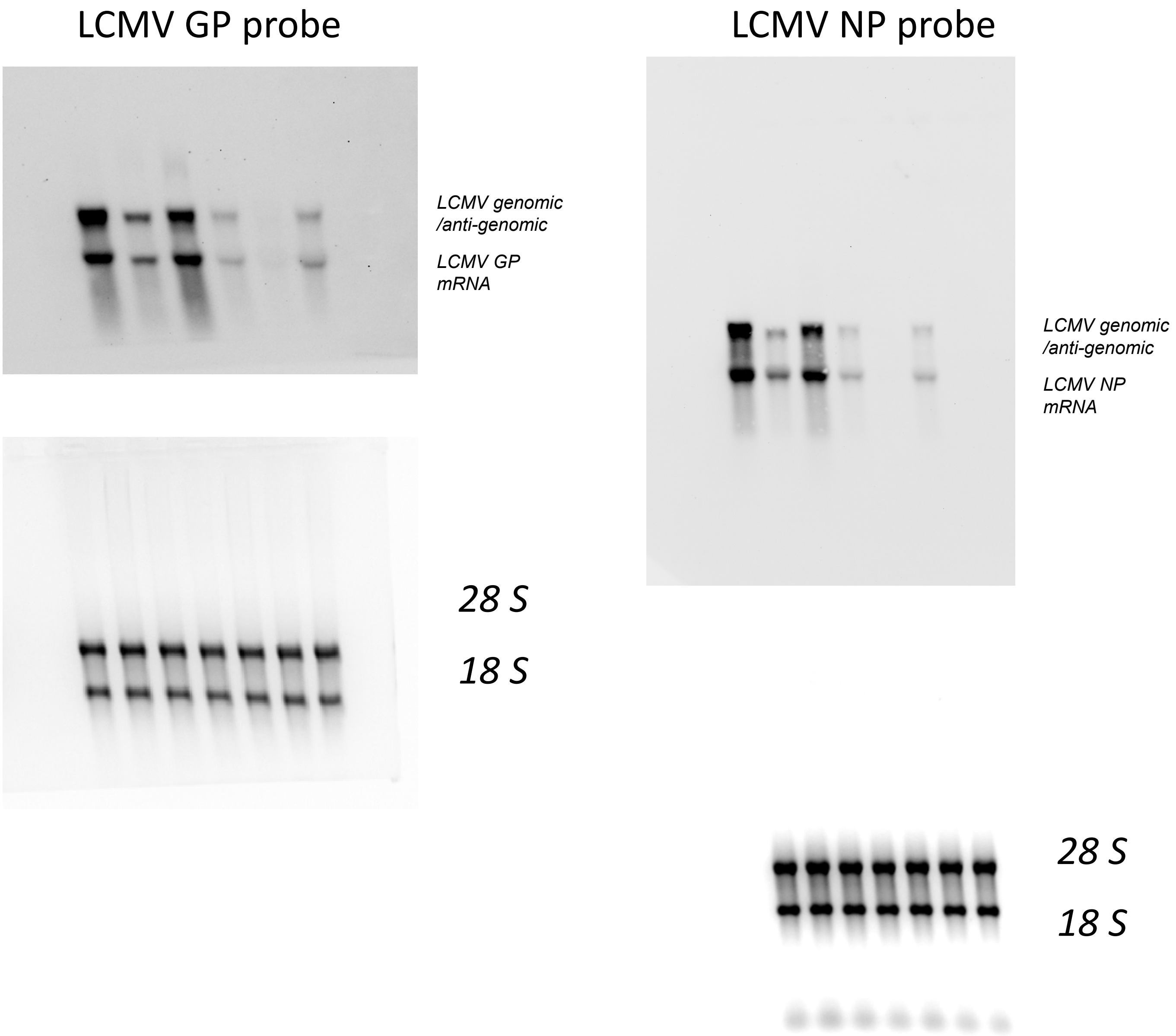


**Supplementary Figure 11: Uncropped scans of Northern blots in Fig. 5h**

LCMV NP staining on BHK21- cells

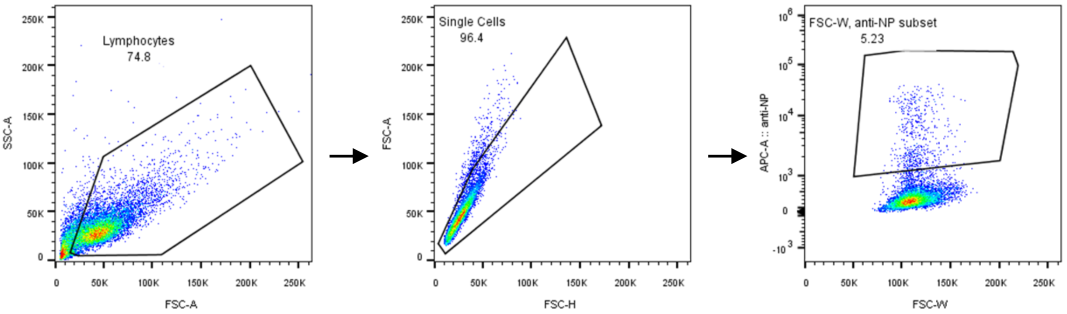


**Supplementary Figure 12: BHK-21 LCMV NP intracellular staining gate strategy of Figure 5k**

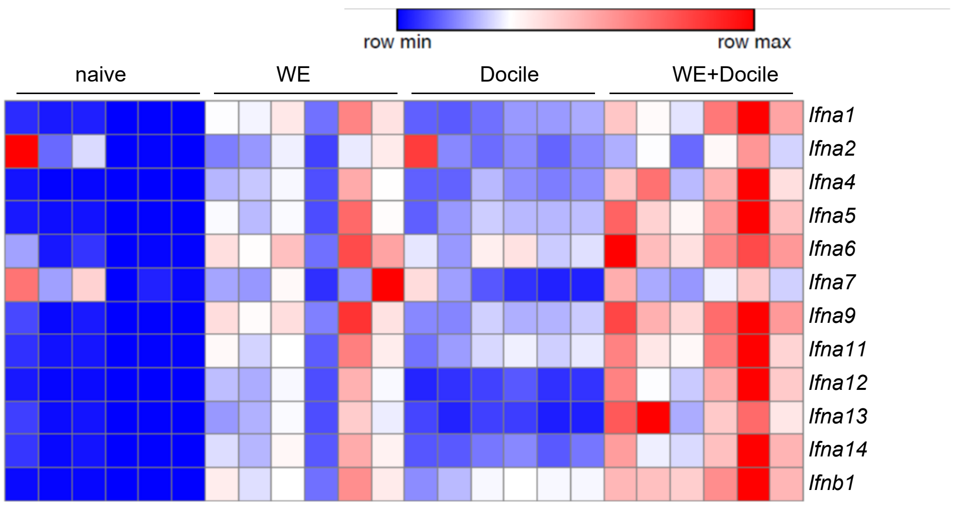


**Supplementary Figure 13: LCMV-WE infection increases IFN-I transcripts.**
C57BL/6 mice were infected with LCMV-WE, LCMV-Docile, or co-infected for 1 day. Various types of IFN-I subtype mRNA transcripts were determined in spleen tissue (n=6).

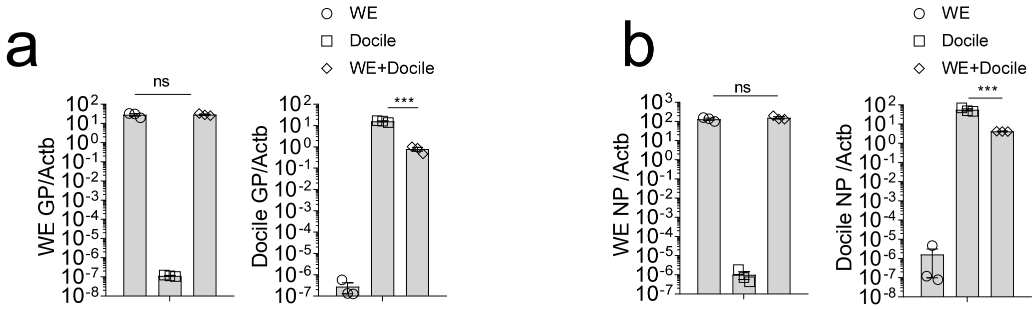


**Supplementary Figure 14: LCMV Docile RNA production was inhibited in the presence of LCMV WE.**

**(a-b)** BHK-21 cells were infected with LCMV-WE, LCMV-Docile, or both at the indicted time points (MOI=1). 24h post-infection, BHK-21 cellular RNA was isolated and **(a)** WE-GP RNA (left panel), Docile-GP RNA (right panel) **(b)** WE-NP RNA (left panel), Docile-NP RNA (right panel) were assessed by RT-PCR (n=3). (Error bars show SEM, ***p < 0.001, and ns indicates statistically not significant between the indicated groups).

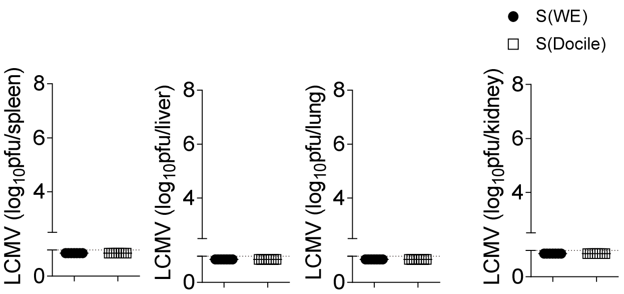


**Supplementary Figure 15: Chimeric LCMV virus is attenuated in WT animals**.
C57BL/6 mice were infected with $2x10^5$ pfu of chimeric virus S(WE)/L(Clone 13) or S (Docile)/L(Clone 13). At day 12 post-infection, virus titers were determined in spleen, liver, lung, and kidney tissue (n=7-8).
