# Supplementary material for: Slow viral propagation during initial phase of infection leads to viral persistence in mice
Source: Commun Biol. 2021 Apr 29;4:508. doi: 10.1038/s42003-021-02028-x (PMC8084999; doi:10.1038/s42003-021-02028-x)
Supplement: Supplementary file 2 — Description of Additional Supplementary Files [file 42003_2021_2028_MOESM2_ESM.pdf]

Description of Additional Supplementary Files

File name: Supplementary Data 1

Description: Source data for the main figures.
