# Supplementary material for: Slow viral propagation during initial phase of infection leads to viral persistence in mice
Source: Commun Biol. 2021 Apr 29;4:508. doi: 10.1038/s42003-021-02028-x (PMC8084999; doi:10.1038/s42003-021-02028-x)
Supplement: Supplementary file 4 — Reporting Summary [file 42003_2021_2028_MOESM4_ESM.pdf]

nature research

Corresponding author(s): Philipp A. Lang

Last updated by author(s): Mar 8, 2021

Reporting Summary

Nature Research wishes to improve the reproducibility of the work that we publish. This form provides structure for consistency and transparency in reporting. For further information on Nature Research policies, see our Editorial Policies and the Editorial Policy Checklist.

Statistics

For all statistical analyses, confirm that the following items are present in the figure legend, table legend, main text, or Methods section.

n/a	Confirmed	
☐	☒	The exact sample size (*n*) for each experimental group/condition, given as a discrete number and unit of measurement
☐	☒	A statement on whether measurements were taken from distinct samples or whether the same sample was measured repeatedly
☐	☒	The statistical test(s) used AND whether they are one- or two-sided<br>*Only common tests should be described solely by name; describe more complex techniques in the Methods section.*
☒	☐	A description of all covariates tested
☐	☒	A description of any assumptions or corrections, such as tests of normality and adjustment for multiple comparisons
☐	☒	A full description of the statistical parameters including central tendency (e.g. means) or other basic estimates (e.g. regression coefficient) AND variation (e.g. standard deviation) or associated estimates of uncertainty (e.g. confidence intervals)
☐	☒	For null hypothesis testing, the test statistic (e.g. *F*, *t*, *r*) with confidence intervals, effect sizes, degrees of freedom and *P* value noted<br>*Give P values as exact values whenever suitable.*
☒	☐	For Bayesian analysis, information on the choice of priors and Markov chain Monte Carlo settings
☒	☐	For hierarchical and complex designs, identification of the appropriate level for tests and full reporting of outcomes
☒	☐	Estimates of effect sizes (e.g. Cohen's *d*, Pearson's *r*), indicating how they were calculated

Our web collection on statistics for biologists contains articles on many of the points above.

Software and code

Policy information about availability of computer code

Data collection	LI-COR Odyssey Infrared Imaging System (Western Blot), BD FACS DIVA of BD Fortessa (Flow cytometry), Prism Genetic Analyzer 3130-16 Applied Biosystems (DNA sequencing), Lumi-Imager™ F1 INTAS (Northen Blot), QuantStudio Real-Time PCR system
Data analysis	Image J Software, Graph Pad Prism, BioEdit, Flow Jo Software

For manuscripts utilizing custom algorithms or software that are central to the research but not yet described in published literature, software must be made available to editors and reviewers. We strongly encourage code deposition in a community repository (e.g. GitHub). See the Nature Research guidelines for submitting code & software for further information.

Data

Policy information about availability of data

All manuscripts must include a data availability statement. This statement should provide the following information, where applicable:

- Accession codes, unique identifiers, or web links for publicly available datasets
- A list of figures that have associated raw data
- A description of any restrictions on data availability

The authors declare that all data supporting the fingdings of this study are avaible within the article and its Supplementary Information files, or are available from the authors upon request.

Field-specific reporting

Please select the one below that is the best fit for your research. If you are not sure, read the appropriate sections before making your selection.

☒ Life sciences ☐ Behavioural & social sciences ☐ Ecological, evolutionary & environmental sciences

For a reference copy of the document with all sections, see nature.com/documents/nr-reporting-summary-flat.pdf

Life sciences study design

All studies must disclose on these points even when the disclosure is negative.

Sample size	No statistical method to determine sample size was performed. Number of animals per group was chosen based on previous experiments with similar experimental set-up.
Data exclusions	No data was excluded from the analysis.
Replication	Experiments were performed with at least three different biological replicates. Attempts at replication were successful.
Randomization	Mice were randomized prior to each experiment
Blinding	Investigators were not blinded for the in vivo experiments as the experiments were carried out by one person. However some key samples i.e. titer, serum cytokines were analyzed by different person without knowledge of the sample ID

Reporting for specific materials, systems and methods

We require information from authors about some types of materials, experimental systems and methods used in many studies. Here, indicate whether each material, system or method listed is relevant to your study. If you are not sure if a list item applies to your research, read the appropriate section before selecting a response.

Materials & experimental systems

n/a	Involved in the study	
☐	☒	Antibodies
☐	☒	Eukaryotic cell lines
☒	☐	Palaeontology and archaeology
☐	☒	Animals and other organisms
☒	☐	Human research participants
☒	☐	Clinical data
☒	☐	Dual use research of concern

Methods

n/a	Involved in the study	
☒	☐	ChIP-seq
☐	☒	Flow cytometry
☒	☐	MRI-based neuroimaging

Antibodies

Antibodies used

FITC-IL7R Invitrogen 11-1271-85 Clone: A7R34 Dilution factor: 1:300
PE-CF594 -PD-1 Biosciences 562523 Clone: J43 Dilution factor: 1:300
PE-TIM3 Invitrogen 12-5870-82 Clone: RMT3-23 Dilution factor: 1:300
BV711-Lag3 Biosciences 563179 Clone: C9B7W Dilution factor: 1:300
BV510-CD62L Biosciences 563117 Clone: MEL-14 Dilution factor: 1:300
PE-cy7-CXCR5 Biosciences 560617 Clone: 2G8 Dilution factor: 1:300
BV786-CD44 Biosciences 563736 Clone: IM7 Dilution factor: 1:300
eFluor 450-KLRG1 Invitrogen 48-5893-82 Clone: 2F1 Dilution factor: 1:300
FITC-SiglecH Invitrogen 11-0333-82 Clone: eBio440c Dilution factor: 1:300
PE-F4/80 Invitrogen 12-4801-82 Clone: BM8 Dilution factor: 1:300
PE-cy7-CD11c Invitrogen 25-0114-82 Clone: N418 Dilution factor: 1:300
APC-B220 Invitrogen 17-0452-82 Clone: RA3-6B2 Dilution factor: 1:300
Alexa Fluor 700-MHCII Invitrogen 56-5321-82 Clone: M5/114.15.2 Dilution factor: 1:300
BV421-CD40 Biosciences 562846 Clone: 3/23 Dilution factor: 1:300
BV510-CD86 Biosciences 563077 Clone: GL1 Dilution factor: 1:300
BV605-CD11b Biosciences 563015 Clone: M1/70 Dilution factor: 1:300
BV650-CD80 Biosciences 563687 Clone: 16-10A1 Dilution factor: 1:300
BV711-CD8 Biosciences 563046 Clone: 53-6.7 Dilution factor: 1:300
BV786-CD19 Biosciences 563333 Clone: 1D3 Dilution factor: 1:300
FITC-CD40 Invitrogen 11-0402-82 Clone: HM40-3 Dilution factor: 1:300

PE-CD86 Invitrogen 12-0862-82 Clone: GL1 Dilution factor: 1:300
APC-CD80 Invitrogen 17-0801-82 Clone: 16-10A1 Dilution factor: 1:300
APC-eFluor 780-MHCII Invitrogen 47-5321-82 Clone: M5/114.15.2 Dilution factor: 1:300
PerCP-eFluor 710-CD8 Invitrogen 46-0081-82 Clone: 53-6.7 Dilution factor: 1:300
PerCP-eFluor 710-CD4 Invitrogen 46-0041-82 Clone: GK1.5 Dilution factor: 1:300
APC-IFN gamma Invitrogen 17-7311-82 Clone: XMG1.2 Dilution factor: 1:300
eFluor 450-TNF alpha Invitrogen 48-7321-82 Clone: MP6-XT22 Dilution factor: 1:300
PerCP-Cyanine5.5-IL2 Invitrogen 45-7021-82 Clone: JES6-5H4 Dilution factor: 1:300
PE-CD8 Invitrogen 12-0083-82 Clone: eBioH35-17.2 Dilution factor: 1:300
PE-CD4 Invitrogen 12-0041-82 Clone: GK1.5 Dilution factor: 1:300
APC-Donkey Anti-Rat IgG (H+L) Jacksonimmuno 712-136-150 Polyclonal Dilution factor: 1:200
Unlabelled Rat anti-LCMV NP Made in house N/A VL-4 Dilution factor: 1:100
RIG-I (D14G6) antibody Cell Signaling Technology Cat #3743
MDA-5 (D74E4) antibody Cell Signaling Technology Cat #5321
MAVS(C-1) Santa Cruz Cat #365333
Phospho-TBK1/NAK(Ser172) (D52C2) antibody Cell Signaling Technology Cat #5483
Phospho-IKKε (Ser172) (D1B7) Cell Signaling Technology Cat #8766
TBK1/NAK antibody Cell Signaling Technology Cat #3013
IKKε antibody Cell Signaling Technology Cat #2690
Phospho-IRF-7 (Ser437/438) (D6M2I) antibody Cell Signaling Technology Cat #24129
IRF-7 antibody Abcam Cat #ab109255
Phospho-IκBα (Ser32/36) (5A5) antibody Cell Signaling Technology Cat #9246
IκBα (44D4) antibody Cell Signaling Technology Cat #4812
Phospho-p44/42 MAPK (Erk1/2) (Thr202/Tyr204) (E10) antibody Cell Signaling Technology Cat #9106
p44/42 MAPK (Erk1/2) (137F5) antibody Cell Signaling Technology Cat #4695
Phospho-SAPK/JNK (Thr183/Tyr185) (81E11) antibody Cell Signaling Technology Cat #4668
SAPK/JNK antibody Cell Signaling Technology Cat #9252
IL-1β (D6D6T) antibody Cell Signaling Technology Cat #31202
Phospho-Stat1 (Tyr701) (58D6) antibody Cell Signaling Technology Cat #9167
Stat1antibody Cell Signaling Technology Cat #9172
β-Tubulin (9F3) antibody Cell Signaling Technology Cat #5346

Validation	We use the manufactures' validated antibodies and available information can be inferred form the catalog number and found on websites.

Eukaryotic cell lines

Policy information about cell lines

Cell line source(s)	BHK-21 [C-13] (ATCC® CCL-10™), MC57G (ATCC® CRL-2295™) were from ATCC, L929 and VL4 hybridoma were provided by Dr. Karl Lang, Essen, Germany)
Authentication	Cell lines used were not authenticated
Mycoplasma contamination	BHK-21, MC57G and L929 cell line were tested negative for mycoplasma. VL4 hybridoma was not tested for mycoplasma
Commonly misidentified lines (See ICLAC register)	No commonly misidentified cell lines were used

Animals and other organisms

Policy information about studies involving animals; ARRIVE guidelines recommended for reporting animal research

Laboratory animals	Mice were on a C57BL/6J genetic background, 6-20 weeks old
Wild animals	No Wild animals used in this study
Field-collected samples	The study did not involve samples collectedd from the field
Ethics oversight	Experiments were performed under the authorization of LANUV in accordance with German law for animal protection.

Note that full information on the approval of the study protocol must also be provided in the manuscript.

Flow Cytometry

Plots

Confirm that:

- [x] The axis labels state the marker and fluorochrome used (e.g. CD4-FITC).
- [x] The axis scales are clearly visible. Include numbers along axes only for bottom left plot of group (a 'group' is an analysis of identical markers).
- [x] All plots are contour plots with outliers or pseudocolor plots.
- [x] A numerical value for number of cells or percentage (with statistics) is provided.

Methodology

Sample preparation	For tetramer staining: single suspended cells were incuated with tet-gp33 (15 minutes), tet-np396 (15 minutes) or tet-gp61 (30 minutes) at 37°C. Followed by surface antibody staining for 30 minutes at 4°C. Blood samples were lysed with BD RBC lysis Buffer before FACS.<br>For Intracellular cytokine staining, samples were incubated with CD8 or CD4 antibody overnight at 4°C. Then samples were fixed with 2% Formalin in PBS at room temperature for 10 minutes, followed by 2 times washing with Saponin containing FACS buffer (1%FCS, 5mM EDTA in PBS), then samples were incubated with IFN-g, TNFa antibodies for 30 minutes before FACS<br>For DC staining, single cells were incubauted with FC block (1:25 dilution) for 10 minutes at 4°C followed by surface antibodies staining for 30 minutes at 4°C. Viability dye such as 7-AAD were added prior to FACS<br>For LCMV-NP staining, cells were fixed with 2% Formalin in PBS at room temperature for 10 minutes, followed by 2 times washing with Saponin containing FACS buffer, then cells with incubated with anti-LCMV NP (VL4, 1:10 dilution) for 30 minutes at 4°C. Cells were washed again with Saponin containing FACS buffer, followed by incubation with Donkey anti-Rat secondary antibodies for 30 minutes at room temperature.
Instrument	BD Fortessa
Software	BD FACSDiva Software was used for collecting and analyzing the flow cytometry data. Flowjo was also used for analyzing data.
Cell population abundance	Not Applicable
Gating strategy	General: Leukocytes (FCS-A, SSC-A), doublets exclusion (FSC-H, FSC-A).<br>For tetramer: tet and CD8 double positive, CD19 negative<br>For shot lived effector cells (SLEC): after tetramer gating, IL7R negative and KLRG1 positive, for memory precursor cells (MEPC) after tetramer gating IL7R postive and KLRG1 negative)<br>For ICS: IFNg or TNFa and CD8 or CD4 double positive<br>For primary cDC: pregate on CD19 negative and 7-AAD negative, followed by gating on MHC-II and CD11c postive.<br>For primary pDC: pregate on 7-AAD negative and B220 positive, followed by gateing on Siglec-H postive and CD11c intermediate<br>For BMDC: 7-AAD negative and CD11c postive<br>For LCMV NP: pregate on BHK-21 cells followed by gating on LCNV-NP postive cells

- [x] Tick this box to confirm that a figure exemplifying the gating strategy is provided in the Supplementary Information.
